# A state-of-the-art systematic review of cancer in hidradenitis suppurativa

**DOI:** 10.1080/07853890.2024.2382372

**Published:** 2024-07-24

**Authors:** Nessr Abu Rached, Jonas Rüth, Thilo Gambichler, Lennart Ocker, Falk G. Bechara

**Affiliations:** a International Centre for Hidradenitis suppurativa/Acne inversa (ICH), Department of Dermatology, Venereology and Allergology, Ruhr-University, Bochum, Germany; b Skin Cancer Centre, Department of Dermatology, Venereology and Allergology, Ruhr-University Bochum, Bochum, Germany; c Department of Dermatology and Phlebology, Christian Hospital Unna, Unna, Germany; d Department of Dermatology, Dortmund Hospital gGmbH and Faculty of Health, Witten/Herdecke University, Dortmund, Germany

**Keywords:** Hidradenitis suppurativa, HS, malignant diseases, lymphoma, colorectal carcinoma, oropharyngeal carcinoma, cutaneous squamous cell carcinoma, malignancy

## Abstract

**Purpose:**

Hidradenitis suppurativa (HS) is a chronic inflammatory disease associated with an increased risk of malignancy. The aim of this systematic review was to investigate the prevalence of different malignancies in HS.

**Methods:**

This review meets the PRISMA criteria. A data-driven approach was used to conduct the research, which involved a detailed keyword search. The study considered meta-analyses, experimental studies, case-control studies, cross-sectional studies, cohort studies, and recently published cases, published in English or German. Excluded were reviews, summaries, and letters to the editor, as well as studies, which are not based on the human population.

**Results:**

Out of the initial 443 publications found, 25 met the inclusion criteria for this systematic review. Patients with HS have a significantly increased risk of cancer, up to 50%. Additionally, the risk of oropharyngeal, central nervous system, colorectal, prostate, vulvar and non-melanocytic skin cancers increase with the severity of HS. The likelihood of comorbid lymphoma in patients with HS is significantly higher compared to healthy controls. In severe cases of HS, malignant degeneration of lesions in the groin, perianal, perineal, and gluteal region can occur in up to 4.6% of cases. This leads to the development of cSCC, which often have a complicated course, are more refractory to treatment and associated with a poorer outcome. The pathogenic mechanisms responsible for the malignant transformation of HS are currently unknown.

**Conclusions:**

Patients with HS have a higher risk of cancer compared to the general population. Untreated, long-standing HS lesions can lead to complicated malignant degeneration resulting in cutaneous squamous cell carcinoma. The mechanisms underlying this malignant degeneration are not fully understood. HS patients also have an increased risk of developing other cancers, including prostate, oral, pharyngeal and colorectal cancers of the central nervous system and lymphomas.

## 1. Introduction

Hidradenitis suppurativa (HS) is a chronically relapsing inflammatory skin disease that predominantly affects the axillary, submammary, genital, inguinal, and perianal regions. Characteristically, a patient with HS develops inflammatory nodules, abscesses, sinuses, fistulas, scars, and contractures [1]. The global prevalence rate of HS is 0.4–1% [2,3]. In the Caucasian population, the ratio of women to men is about 3:1 [4]. According to the Hurley classification system, which is commonly used to categorise the clinical severity of HS, stage 1 is defined as non-scarring, transient abscesses without sinus tracts, stage 2 is specified as the formation of a single sinus tract in recurrent abscesses, and stage 3 is designated as multiple abscesses, and fistulae with extensive scarring [5]. Treatment options for HS include topical antiseptics, local and systemic antibiotics, high-dose zinc therapy and surgery. The systemic treatment with Adalimumab or Secukinumab in moderate to severe HS offers new promising non-surgical treatment approaches. Further, studies on the treatment of HS with other biologics and small molecules are currently being conducted. Nevertheless, a combined approach involving both systemic and surgical treatments remains the standard treatment for patients with moderate to severe HS, and may be beneficial [6]. Currently, the pathogenesis of HS remains partially elusive; however, it is presumed to be multifactorial in nature, with a genetic predisposition. HS is linked with smoking, type II diabetes mellitus, polycystic ovary syndrome (PCOS), metabolic syndrome, obesity, dyslipidaemia, hypertension, inflammatory bowel disease, spondyloarthropathy, and psychiatric comorbidity such as depression [7–12].

An association between HS and malignancy is still controversial. A meta-analysis conducted by Bailey et al. showed an increased risk of developing cutaneous squamous cell carcinoma (cSCC) (RR = 2.67, *p* < 0.001), oral and pharyngeal cancer (*p* < 0.001) and malignant lymphoma (*p* < 0.001), as well as an increased overall risk of cancer (RR = 1.30, *p* < 0.001) [13]. Population-based cohort from Sweden and South Korea also confirmed these associations [14,15]. Numerous case reports and case series on the occurrence of non-melanoma skin cancer (NMSC) in patients with HS can be found in the literature. The development of cSCC is often described as a complication of long-standing, advanced HS disease, primarily in men [16,17]. However, evidence on the occurrence of malignancies in HS patients is lacking. Therefore, this study aims to combine information on the pathogenesis of HS with a review of the evidence on HS and malignancy.

## 2. Methods

### 2.1. Search strategy

**Figure 1.**
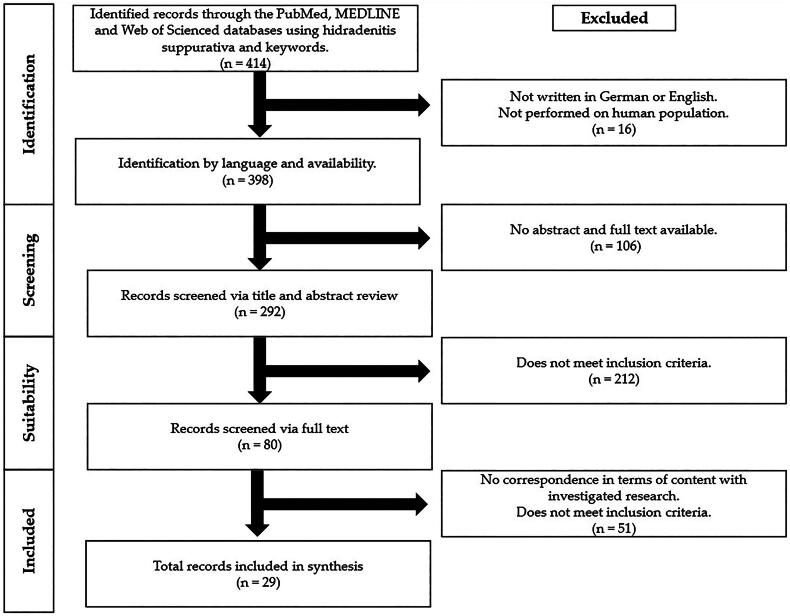
Flowchart with the research strategy of this systematic review.

In this systemic review, we conducted a search in PubMed, Medline and Web of Science search in January 2024 according to the 2020 PRISMA criteria using the following search terms [18]: (“hidradenitis suppurativa” OR “acne inversa”) AND (“malignant disease” OR “cancer” OR “carcinoma” OR “neoplasm” OR “neoplasia” OR “skin cancer” OR “melanoma” OR “marjolin ulcer” OR “leukemia” OR “AIDS-related cancers” OR “sarcoma” OR “tumors” OR “lymphoma” OR “metastases” OR “metastasis” OR “mycosis fungoides” OR “sezary-syndrom” OR “chemotherapy” OR “radiation” OR “immunotherapy” OR “tumor surgery” OR “hematological diseases” OR “overall cancer risk” OR “cancer incidence”). We identified 444 papers published between 1978 and 2024. Figure 1 shows the flowchart of the search strategy.

### 2.2. Eligibility criteria

Meta-analyses, experimental studies, case-control studies, cross-sectional studies, cohort studies, and recently published case reports and series were included if they were published in English or German and related to the human population. Reviews, abstracts and letters to the editor were excluded from this review. Eligible articles were searched and selected by two medical scientists. An additional scientist was consulted in cases of uncertainty about inclusion and exclusion. Articles were assessed for eligibility by screening their titles and abstracts. The full texts were then assessed to determine the level of evidence based on the type of study in each article. Additionally, we searched for ongoing clinical trials using “HS” and “Hidradenitis suppurativa” as keyword in the library of clinical trials, which is available on https://clinicaltrials.gov/.

### 2.3. Quality assessment

The level of evidence for each of the identified articles was determined after examining the study methodology using the GRADE scale [19]. The systematic review included 14 case reports (level of evidence V), 2 cross-sectional studies (level of evidence IV), 1 case-control study (level of evidence IV), 6 cohort studies (level of evidence III), and 2 meta-analyses (level of evidence I).

## 3. Results

A total of 25 studies were included in this review. The included articles dealt with 3 articles on overall cancer risk, 9 articles on NMSC, 8 articles about vulvar and 1 article on cervix cancer, 10 articles on lymphoma and 3 articles on various malignancies. The search of the Clinicaltrials.gov databank did not yield any additional studies.

### 3.1. Overall cancer risk in patients with HS

**Figure 2.**
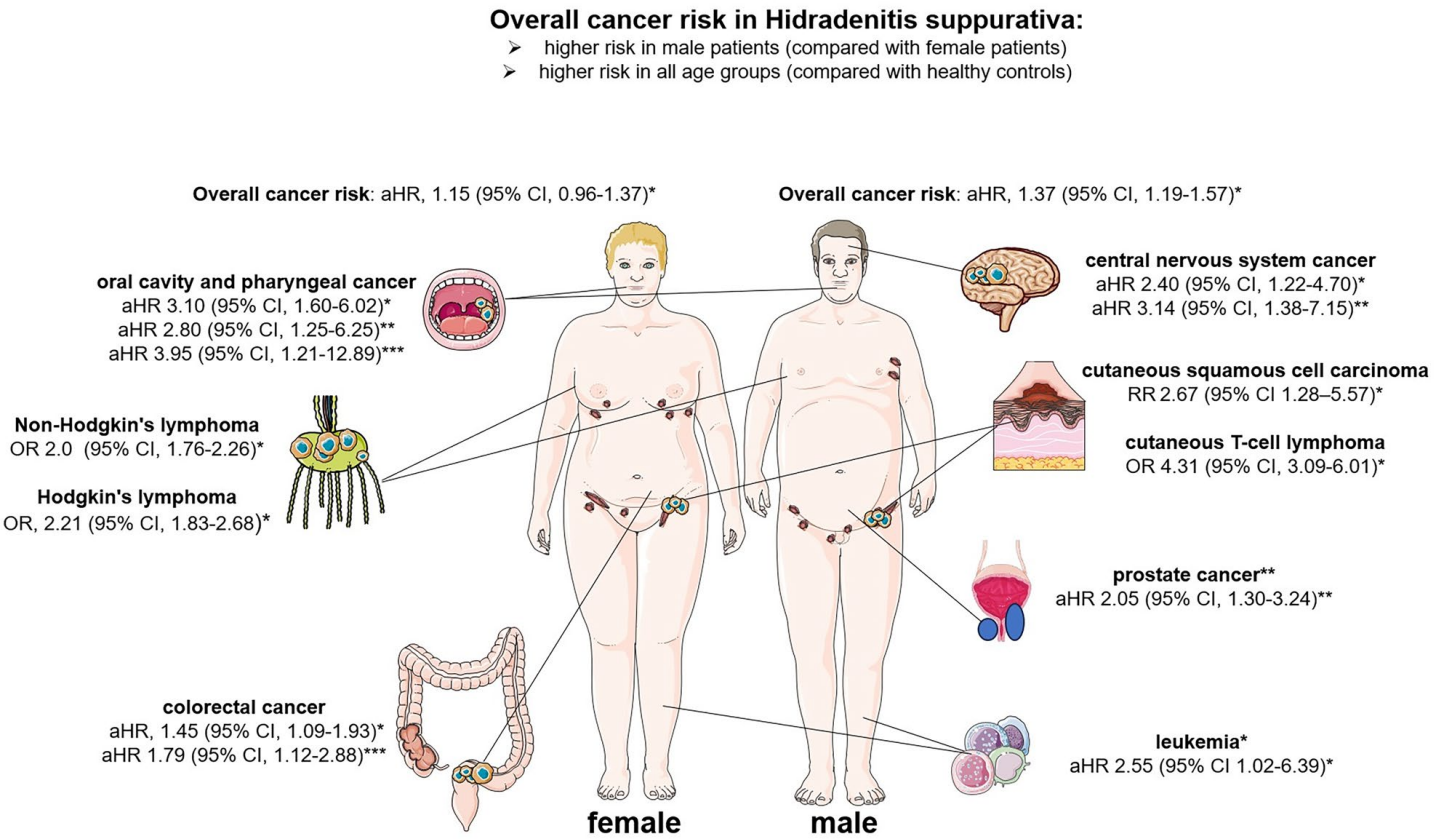
Shows the different ratios and the overall cancer risk in comparison; or, odds ratio; RR: relative risk; aHR, adjusted hazard ratio; *Compared to healthy controls; **Compared to healthy male controls; ***compared to healthy female controls.

An association between HS and the development of certain cancers has been reported [7]. This review includes two cohort studies and a meta-analysis examining the overall cancer risk in HS patients (Table 1 and Figure 2: HS and overall cancer risk). A retrospective, population-based cohort study was conducted on 2119 Swedish HS patients from 1965 to 1997, with a mean follow-up time of 9.8 years. During this period, there were a total of 81 cancer cases occurred (standardized incidence ratio (SIR) 1.9; 95% CI, 0.8-3.8). Compared with to an age- and sex-matched healthy group, the risk of developing any cancer was observed to be 50% higher. Men had a higher risk of developing cancer in comparison to women. The incidence of all-site cancer significant increase in individuals with comorbid diabetes mellitus (*p* = 0.01) [14]. Whereas alcoholism and surgical history were not associated with an increased risk of cancer. After excluding HS patients with diabetes mellitus, statistical analysis still showed a significantly increase in cancer cases (*p* < 0.01) [14]. A population-based cohort study analysed 22,468 HS patients and 179,734 matched controls from 2007 to 2018 in Korea. After adjustment for the presence of hypertension and dyslipidaemia, a significant increase in cancer risk was observed in the HS cohort (adjusted HR (aHR), 1.28; 95% CI, 1.15-1.42). In men, there was a significant increase in cancer risk compared with the healthy control group (aHR, 1.37; 95% CI, 1.19-1.57). However, no significant difference was observed among female patients (aHR, 1.15; 95% CI, 0.96-1.37). Furthermore, the aHR increased in younger patients (<40 years: aHR, 1.34; 95% CI, 1.06-1.68) as well as older patients with HS (≥40 years: aHR, 1.26; 95% CI, 1.11-1.42). Analysing a subset of the HS population showed that patients with severe HS disease had a significantly higher aHR (1.49; 95% CI, 1.15-1.92) compared to those with mild disease (aHR, 1.24; 95% CI, 1.11-1.40). In addition, the subgroup analysis showed an increased incidence rate in patients with HS who were receiving systemic therapy with TNF-α inhibitors (1030 cases per 100,000 patient-years) [15]. In 2023, a meta-analysis of two cohort and four case-control studies was performed by Bailey et al. Their qualitative analysis suggested a significant increase in the relative risk (RR) of cancer in HS patients (RR = 1.30, 95%CI 1.18-1.43, *p* < 0.001, I2 = 0%) [13].

**Table 1.** HS and overall cancer risk.

References	Type of source (*Level of evidence*)	Study population	Results	Conclusion
Lapins et al. 2001 [14]	Retrospective, population-based cohort study (*III*)	*n* = 2119 (1495 female and 624 male)	81 total cases of cancer (52 female and 29 male) in patients with HS. Patients with HS had an 50% increased risk for any cancer. There were overrepresented cancer cases in HS patients with diabetes mellitus.	There is a 50% increased risk of developing malignant neoplasms in HS. An association between cancer in HS patients’ diabetes mellitus, bacterial colonization and chronic inflammation appears to exist.
Jung et al. 2021 [15]	Retrospective, population-based cohort study (*III*)	*n* = 202202 HS cohort *n* = 22468 (8161 female and 14307 male) Matched control cohort *n* = 179734 (65228 female and 114466 male)	Hypertension (p < 0.001), type 1 diabetes (p< 0.001), type 2 diabetes (p < 0.001), dyslipidemia (p < 0.001), alcoholic liver disease (p= 0.008), chronic obstructive pulmonary disease (p = 0.03), chronic kidney disease (p < 0.001), and heart failure (p = 0.01) were found to be more common in patients with HS than in healthy controls. For comorbidities, aHR showed a significantly higher overall risk of cancer in patients with HS than in controls (aHR, 1.28; 95% CI, 1.15-1.42). Overall cancer risk was higher in male patients with HS than in male controls (aHR, 1.37; 95% CI, 1.19-1.57). Overall cancer risk was not higher in female patients with HS than in the female controls (aHR, 1.15; 95% CI, 0.96-1.37). Overall cancer risk was increased regardless of HS patients’ age (age <40 years: aHR, 1.34; 95% CI, 1.06-1.68; age ≥40 years: aHR, 1.26; 95% CI, 1.11-1.42).	The overall risk of cancer in male patients with HS is significantly increased in all age cohorts. In contrast, the cancer risk in female patients with HS isn’t significantly higher.
Bailey et al. 2023 [13]	Meta-Analysis: 6 included Studies (*I*)		Patients with HS had a significantly increased overall cancer risk (RR = 1.30, 95%CI 1.18–1.43, *p* < 0.001, I2 = 0%).	The overall risk of cancer in patients with HS is significantly increased.

HS, Hidradenitis suppurativa; aHR, adjusted hazard ratio; RR, relative risk; CI, confidence interval.

### 3.2. HS and skin cancers

Among malignant skin tumours, a distinction is made between melanoma and non-melanoma skin cancer (NMSC). Numerous publications have described the development of cSCC as a complication of long-standing HS [16,17]. After basal cell carcinoma (BCC), cSCC is the second most common type of NMSC and its incidence is increasing worldwide. In addition to cumulative sun exposure, immunosuppression and persistent inflammation have been shown to be favourable factors for the development of cSCC. In addition, scarring from burns, wounds and ulcers, as well as HPV infection, increase the likelihood of developing cSCC. Actinic keratoses (AK) are a precursor lesion of cSCC [18]. Marjolin’s ulcer is a rare malignant skin tumour that occurs in damaged skin, long-standing scars and chronic wounds [20]. The most common histological subtype of Marjolin’s ulcer is the squamous cell carcinoma (SCC) (80-90%), followed by BCC (9.6%) [14]. The prevalence is higher in men than in women (3:1) [14]. Compared to other forms of NMSC, Marjolin’s ulcer is characterised by aggressive growth, rapid metastatic tendency, high recurrence rate and poorer prognosis [14,15]. This form of malignant degeneration has been observed in secondary healing burns, traumatic wounds, osteomyelitis, pressure ulcers, venous insufficiency, radiation dermatitis, puncture and bite wounds and HS [21–23].

Large cohort and cross-sectional studies as well as meta-analyses showed an increased incidence and risk elevation of NMSC in HS patients [13,14,17,24,25]. In particular, cases of cSCC and Marjolin ulcers developing from HS are reported in the literature [13,14,17, 24–35]. Compared to the other studies (Table 2: HS and NMSC), only one cross-sectional study by Brown et al. detected significantly increased rates for comorbid SCC (OR, 15.70; 95% CI, 10.47-23.55; *p* < 0.001) and BCC (OR, 3.22; 95% CI, 1.21-8.60; *p* < 0.04) [21]. An association with an increased incidence of melanoma in HS patients was not shown in either cohort studies or cross-sectional studies [14,25].

**Table 2.** HS and NMSC.

References	Type of source (*Level of evidence*)	Study population	Results	Conclusion
Ritz et al. 1998 [26]	Retrospective cohort study (*III*)	*n* = 31 (8 female and 23 male)	In the HS cohort, 1 male patient (3.22%) was identified with cSCC based on long-standing genitoanal HS, multiple prior operations and a fatal outcome.	cSCC is a rare complication of a long-standing HS.
Lapins et al. 2001 [14]	Retrospective, population-based cohort study (*III*)	*n* = 2119 (1495 female and 624 male)	HS patients showed a marked increase in the risk of NMSC, particularly cSCC (5 cases; SIR, 4.6; 95% CI, 1.5-10.7). No instances of BCC or AK were detected. HS patients with diabetes mellitus were overrepresented in the cases, but the excess was still evident after excluding those patients.	Patients with HS have a notably heightened susceptibility to NMSC, particularly when associated with smoking, alcohol abuse, and preexisting diabetes mellitus.
Lavogiez et al. 2010 [27]	Retrospective cohort study (*III*)	*n* = 13; all male	The prevalence of cSCC among HS was 4.6%. Malignant transformation of HS into a SCC affects mainly men with a long-term history of genitoanal HS. 8 patients (61.54%) had HPV infection, and all had at least 1 high-risk genotype (HPV 16, 18, and 68). All of them were HPV16 DNA positive. 2 patients (15.38%) concomitantly harbored a low-risk genotype, HPV 6.	HPV infection is involved in the malignant transformation of long-term genitoanal HS.
Shlyankevich et al. 2014 [24]	Retrospective case-control-study (*III*)	*n* = 2292 HS cohort *n* = 1776 (1296 female and 480 male) Control cohort *n* = 1730 (1270 female and 460 male)	Smoking, arthropathies, dyslipidemia, PCOS, psychiatric disorders, obesity, drug dependence, hypertension, diabetes, thyroid disease, alcohol dependence, and lymphoma were significantly associated with HS (all p < 0.01). cSCC in HS was more common in patients with HS than in healthy controls (9 patients in the HS group compared to none in the control group).	There is a higher risk of cSCC in HS patients.
Matoso et al. 2014 [28]	Retrospective cohort study (*III*)	*n* = 29 (all male)	A cSCC on the basis of long-standing HS with scrotal lymphoedema can complicate the course of the underlying disease.	cSCC of the scrotum is a conceivable complication of HS
Kohorst et al. 2019 [29]	Retrospective cohort study (*III*)	*n* = 12 (3 female and 9 male)	cSCC in HS was more common in male patients with long duration of the disease. cSCC arising based on gluteal, perianal and perineal HS; no occurrence in axillary HS has been observed. Invasive cSCC arising in HS correlated with increased risk for metastasis and fatal outcome (survical rate: 41.7%; recurrence rate: 58,3%; metastasis rate: 16.7%)	Invasive cSCC arising in HS is most common in men with gluteal, perianal, and perineal disease many years after the initial onset of HS and carries a high risk of death
Rancelli et al. 2021 [17]	Meta-Analysis of case reports and series (*I*)	*n* = 138 (36 female and 102 male)	cSCC arising in HS is more common in long-standing HS and more in men than women. cSCC occurring in HS has an overall 5-year survival of 38.5%. The risk of death is correlated with the histologic grade and clinical stage of cSCC: The detection of poorly differentiated cSCC (*p* < 0.0001), presence of nodal metastasis by (*p* < 0.0001), and the presence of distal metastasis (*p* < 0.0001) worsen the outcome. Patients with cSCC surgically excised performed better than those needing radiotherapy and/or polychemotherapy (p < 0.0001). The risk of death is significantly increased if cSCC recurs after treatment either at the excision site (p < 0.05) or at nearby site (p < 0,05). No statistically difference was found for HS severity and duration, disease location, smoking, and/or HPV status.	cSCC arising in HS has an overall 5-year survival of 38.5%. A higher risk of death correlates significantly with histology subtypes, advanced grades and stages, as well as locoregional recurrence.
Brown et al. 2023 [25]	Cross sectional study (*IV*)	*n* = 90879561 HS cohort *n* = 62875 (37985 female and 24890 male) Control cohort *n* = 90816776(51220662 female and 39596113 male)	HS was significantly associated with pyoderma gangrenosum, acne vulgaris, acne conglobata, dissecting cellulitis, cellulitis, impetigo, pilonidal cysts, SCC, psoriasis, atopic dermatitis, prurigo nodularis, lichen simplex chronicus, seborrhoeic dermatitis, pemphigus vulgaris, rosacea and dermatitis (all p < 0.001) as well as BCC (p = 0.04). The prevalence of dermatological diseases mentioned was significantly higher in patients with HS than in health controls (24.60% vs. 5.30%, p < 0.001). Patients with HS had significantly greater rates of comorbid cSCC (p < 0.001) and BCC (p < 0.04) than patients without HS. There were no association between HS and melanoma.	Patients with a diagnosis of HS are more likely to have a concomitant diagnosis of cSCC and BCC, but there wasn’t an association between HS and melanoma.
Bailey et al. (2023) [13]	Meta-Analysis: 6 studies included (*I*)		Patients with HS had significantly increased risk of cSCC (RR = 2.67, 95%CI 1.28–5.57, p < 0.001, I2 = 34%).	There is a higher risk of cSCC in patients with HS.

HS, Hidradenitis suppurativa; NMSC, non-melanoma skin cancer; cSCC, cutaneous squamous cell cancer; BCC, basal cell carcinoma; AK, actinic keratosis; HPV, human papillomavirus; PCOS, polycystic ovary syndrome; RR, relative risk; CI, confidence interval.

The Swedish cohort study by Lapins et al. showed a significant increase in the incidence of NMSC in HS patients (SIR, 4.6; 95% CI, 1.5-10.7). It was observed that cancer cases were disproportionately higher in patients with diabetes mellitus compared to those without (*p* = 0.01) [14]. Even after excluding the patients with diabetes mellitus, there was still a high number of NMSC cases [14]. In a retrospective cohort study by Lavogiez et al. cSCC in lesional HS was found in 13 male subjects out of a total of 217 HS. The prevalence of cutaneous cSCC in patients with HS was 4.6%. The mean age at diagnosis of cSCC was 53 years, while the average time from the initial HS diagnosis to cSCC diagnosis was 25.3 years. Ten of the patients were smokers with more than 30 pack-years. Lymphatic and visceral metastases occurred in 2 and 3 cases, respectively. Three patients showed aggressive local growth with osteolysis. Within the one-year follow-up period, the mortality rate was 23.1% (3 of 13 patients). Histopathological examination of tumour biopsies identified well-differentiated SCC in seven cases and verrucous carcinoma in six cases, accounting for 53.8% and 46.2% respectively. The presence of HPV was tested by PCR in eight anogenital tumour samples, showing the presence of α-HPV with low-risk types (HPV-6), α-HPV with high-risk types (HPV-16 and HPV-68) and β-HPV (HPV types 17, 22, 23, 38, 77, 81) in two, eight and seven cases, respectively. Therefore, the authors suspected the involvement of HPV in the malignant transformation of HS [27]. Rancelli et al. conducted a meta-analysis of 34 published case reports or series detailing 138 cases of SCC that occurred in HS [17]. This study focused on clinical characteristics and determinants predicting severe symptoms. The results showed that men were more likely to develop cSCC than women (OR 1.9; 95% CI, 0.673-5.376, *p* = 0.225). Furthermore, the combined analysis showed a 5-year survival rate of 38.5%. The likelihood of mortality was found to be associated with histological grading and clinical stage. Poorly differentiated cSCC was associated with a 7.2-fold increased risk (*p* < 0.0001), nodal metastasis with a 9.7-fold increased risk (*p* < 0.0001) and the presence of distant metastases with a 4.3-fold increased risk (*p* < 0.0001). Notably, patients with surgically excised cSCC had better outcomes compared to those who required radiotherapy and/or polychemotherapy (OR 8.2, *p* < 0.0001). Locoregional recurrence of cSCC either at the excision site (OR 5.2, *p* < 0.05) or at a nearby location (OR 7.9, *p* < 0.05) significantly increased the risk of death. The researchers found no statistically significant difference in HS severity and duration, location, smoking, and/or HPV status. However, the female gender and white race showed a favourable prognostic trend, although the results were not statistically significant (*p* > 0.05) [17]. Cohort studies suggest an increased risk of developing cSCC, particularly in HS involving the perineal, perianal and gluteal regions [24,27,29]. Male patients are more likely to develop this type of skin cancer. In addition, those whose disease persists for a long time are at increased risk. For example, prolonged disease duration is another factor that increases the likelihood of malignant outcomes in HS [24].

### 3.3. HS and malignant tumours of the female genital system

In addition, there are reports in the literature of malignant transformation of genital HS in women into malignancies of the female genital tract, in addition to the occurrence of SCC in HS lesions. However, the retrospective cohort studies by Lapins et al. and Jung et al. did not find a statistically significant increased risk of developing malignancies of the external female genitalia [14,15]. Eight case reports and one case series (total *n* = 10) were identified regarding the development of vulvar or cervical cancer in chronic genitourinary HS (Table 3: Case reports on malignancies of the female genital system in HS patients). No case reports of vaginal cancer were identified in this systematic review. However, characteristics were not available for all cases. The mean age of the reported cases was 56 years (range: 33-75 years), with a mean duration of HS before cancer diagnosis of 25.4 years (range: 7-30 years). Predominantly, the malignancy cases reported SCC of the vulva (90%). In only one case (10%) was grade III cervical intraepithelial neoplasia (CIN) reported. All, but one case of Hurley stage II (2 cases) or stage III (7 cases). In 7 cases, metastases were noted. The treatments varied and included radiotherapy (20%), radiochemotherapy (20%) and wide surgical excision (60%). PCR analysis was carried out on tumour biopsies to determine the HPV status in two publications, revealing two instances of high-risk HPV types. 40% of the patients died as a result of malignancy.

**Table 3.** Case reports reported malignant tumors of the female genital tract in HS patients (*level of evidence: V*).

Reference (year)	Age (years)	Duration of chronic HS prior carcinoma (years)	Hurley stage	Treatment of HS	Location of carcinoma	Histology type	HPV Status	Metastasis (Y/N)	Tobacco use (Y/N)	Treatment	Outcome
Manolitsas et al. (1999) [30]	52	30	III	Multiple surgical excision	Vulvar	SCC, Grade 1	Unknown	N	Unknown	Wide surgical excision and primary closure.	Alive at the time of publication.
Crain et al. (2005) [31]	44	20	III	Unknown	Right labia, distal vagina and urethra with satellite lesions on the mons and right medial tight	SCC, Grade 2	Unknown	Y	Unknown	Palliative high dose radiation	Died after 6 months.
Short et al. (2005) [32]	57	15	II	No	Vulvar	SCC	Unknown	N	Y	Wide surgical excision.	Alive at the time of publication.
Mac Lean et al. (2007) [33]	61	40	III	Unknown	Vulvar and inguinal lymph nodes	SCC	Unknown	Y	Unknown	Radiotherapy	Died after 2 months.
Pena et al. (2015) [34]	64	Unknown	II	Unknown	Right labia	SCC	Unknown	Y	Unknown	Radiochemotherapy with cisplatin + 5-FU	Alive after 15 months.
Rekawek et al. (2016) [35]	61	Unknown	III	Unknown	Vulvar	SCC	Unknown	Y	Unknown	Radical hemivulvectomy, nodal dissection and endometrial curetting with plastic reconstruction	Alive at the time of publication.
Makris et al. (2017) [36]	52	36	I	Unknown	Right iliac fossa arising from pelvis, right vulvar extending to the groin and left lymph nodes	SCC, Grade 1	Unknown	Y	Unknown	Wide local excision and sentinel lymph node excision	Alive at the time of publication.
Bessaleli et al. (2018) [37]	33	Unknown	Unknown	adalimumab	Cervix	CIN III	Positive for high-risk HPV	N	Unknown	Surgery	Alive at the time of publication.
Vergeldt et al. (2022) [38]	75	>30	III	Systematic antibiotics (clindamycin + rifampicin) (prior multiple surgical incisions)	Vulvar	SCC, Grade 3	Negative	Y	Y	Wide surgical excision and best supportive care	Died shortly after diagnosis.
	61	7	III	adalimumab	Vulvar with ingrowth in anal sphincter, vagina and levator ani muscle with inguinal and pelvic lymphadenopathy	SCC, Grade 2	Positive for high-risk HPV 16	Y	Y	Radiochemotherapy with 1.65 Gy radiation and Cisplatin	Local recurrence within 2 months after finishing treatment. 5 months after posterior exenteration recurrence in the vulvar scar and pelvic floor muscles with possible bone metastases and received palliative chemotherapy. The patient died shortly after this.

Y, yes; N, No; HS, Hidradenitis suppurativa; HPV, human papillomavirus; SCC, squamous cell cancer; CIN, cervical intraepithelial neoplasia; 5-FU, 5-Fluoruracil.

### 3.4. HS and lymphatic neoplasms

Chronic inflammation in HS can lead to the emergence of clonal populations of immune cells that may transform into malignant lymphomas (Table 4: HS and lymphatic neoplasms). A case-control study indicated that the HS group exhibited a prevalence of 2% for lymphoma and a significant association between HS and lymphoma as comorbidity (OR, 3.6; 95% CI, 1.71-7.57; *p* < 0.01) [24]. Cohort studies also demonstrated a significant incidence of haematological cancer cases in HS patients [14,15]. Particularly, they showed an elevated likelihood of developing Hodgkin’s lymphoma (HL), regardless of age and gender variables. Analysis of age-adjusted subgroups showed a greater propensity for leukaemia in younger patients (aHR 2.55; 95% CI 1.02-6.39), whereas the risk of developing Hodgkin’s lymphoma was higher in the older group (aHR 9.04; 95% CI 1.26-64.85) [15]. A cohort study with a cross-sectional design by Tannenbaum et al. examined 62,690 HS patients for the incidence of lymphoma. An increased likelihood of developing non-Hodgkin’s lymphoma (NHL) (OR, 2.00; 95% CI, 1.76-2.26) was observed, including cutaneous T-cell lymphoma (CTCL) (OR, 4.31; 95% CI, 3.09-6.01) and HL (OR, 2.21; 95% CI, 1.83-2.68). Lymphoma was found to be more common in men than in women (*p* = 0.02) [39]. Further subgroup analysis revealed higher rates of NHL and CTCL in patients aged over 40 years compared to younger individuals with HS (aged <40 years) [38]. A meta-analysis validated these results, indicating that patients with HS have an increased risk of developing HL, NHL, and CTCL compared to healthy individuals (all *p* < 0.001) [13]. Case reports of lymphatic neoplasms in patients with HS are presented in Table 5 (Table 6: Cases reported lymphatic neoplasms in HS patients). No case reports on HL and CTCL could be found in the literature search. Lymphomas occurred in younger patients with a brief duration of HS and older patients long after the diagnosis of HS. In three instances, patients were treated with anti-TNF-alpha therapy using adalimumab or infliximab for their HS. In all five cases, complete remission was achieved with stage-appropriate therapy [41,44].

**Table 4.** HS and lymphatic neoplasms.

References	Type of source (*Level of evidence*)	Study population	Results	Conclusion
Lapins et al. 2001 [14]	Retrospective population-based cohort study (*III*)	*n* = 2119 (1495 female and 624 male)	An increased risk for the development of hematopoietic cancers has been noticed.	There is a higher risk of hematopoietic cancers in HS patients.
Shlyankevich et al. 2014 [24]	Retrospective case-control-study (*III*)	*n* = 2292 HS cohort *n* = 1776 (1296 female and 480 male) Control cohort *n* = 1730 (1270 female and 460 male)	Smoking, arthropathies, dyslipidemia, PCOS, psychiatric disorders, obesity, drug dependence, hypertension, diabetes, thyroid disease, alcohol dependence, and lymphoma were significantly associated with HS (all *p* < 0.01). 2% of the HS population showed the diagnosis of lymphoma. There was a significantly association between HS and lymphoma (OR, 3.6; 95% CI, 1.71-7.57).	There is a higher risk of lymphoma in HS patients.
Tannenbaum et al. 2019 [39]	Cross sectional cohort study (*IV*)	HS Cases *n* = 62690 (16030 female and 46660 male)	Patients with HS had increased overall odds of having NHL (OR, 2.00; 95% CI, 1.76-2.26), HL (OR, 2.21; 95% CI, 1.83-2.68), and CTCL (OR, 4.31; 95% CI, 3.09-6.01). Male patients with HS had higher prevalence of NHL, HL and CTCL than in females (p = 0.02). Older patients with HS had higher prevalence of NHL and CTCL.	Patients with HS have an increased risk for having malignant lymphomas.
Jung et al. 2021 [15]	Retrospective, population-based cohort study (*III*)	*n* = 202202 HS-Group *n* = 22468 (8161 female and 14307 male) Matched control cohort *n* = 17973 (65228 female and 114466 male)	Patients with HS were significantly more likely to develop HL (aHR 5.08; 95% CI 1.21-21.36). Compared with younger patients in the control group, younger HS patients had a higher risk of leukemia (aHR 2.55; 95% CI 1.02-6.39). Older HS patients had a higher risk of Hodgkin lymphoma (aHR 9.04; 95% CI 1.26-64.85) compared to their older counterparts in the control group.	There is an elevated risk of developing Hodgkin lymphoma amongst HS patients. Moreover, younger HS patients face an increased risk of developing leukemia.
Bailey et al.; 2023 [13]	Meta-Analysis: 6 studies included (*I*)		Patients with HS had a heightened risk of developing HL, as well as increased odds of acquiring lymphomas such as HL, NHL and CTLC (all p < 0.001).	HS patients have an increased risk for developing lymphomas.

HS, Hidradenitis suppurativa; PCOS, polycystic ovary syndrome; HL, Hodgkin lymphoma; NHL, non-Hodgkin lymphoma; CTCL, cutaneous T-cell lymphoma OR, odds ratio; aHR, adjusted hazard ratio; CI, confidence interval.

**Table 5.** HS and other, various malignant diseases.

References	Type of source (Level of evidence)	Study population	Results	Conclusion
Lapins et al. 2001 [14]	Retrospective, population-based cohort study (*III*)	*n* = 2119; 1495 female and 624 male	Significantly elevated relative risks were observed for buccal cancer (5 cases; SIR, 5.5; 95% CI, 1.8-12.9) and primary liver cancer (3 cases; SIR, 10.0; 95% CI, 2.1-29.2). Not statistically significant, increased risks were noted for esophageal cancer (2 cases; SIR, 7.4; 95% CI, 0.9-26.8), lung cancer (5 cases; SIR, 1.7; 95% CI, 0.6-4.1), kidney cancer (2 cases; SIR, 1.7; 95% CI, 0.2-6.3) and cancers in urinary tract organs other than kidney (3 cases; SIR, 1.9; 95% CI, 0.4-5.5). No obviously increased risk was observed for colon, rectum, breast, female genital system, and brain cancers. There were overrepresented cancer cases in HS patients with diabetes mellitus, alcohol abuse and smoking.	HS patients have an increased risk for oral cavity and primary liver cancer, especially with smoking, alcohol abuse and diabetes mellitus as preexisting conditions.
Jung et al. 2021 [15]	Retrospective, population-based cohort study (*III*)	*n* = 202202 HS-Group *n* = 22 468; 8161 female and 14307 male Matched control cohort *n* = 179734; 65228 female and 114466 male	Patients with HS were at significantly higher risk for developing oral cavity and pharyngeal cancer (aHR, 3.10; 95% CI, 1.60-6.02), central nervous system cancer (aHR, 2.40; 95% CI, 1.22-4.70), prostate cancer (aHR, 2.05; 95% CI, 1.30-3.24), and colorectal cancer (aHR, 1.45; 95% CI, 1.09-1.93). Female HS patients had a higher risk of oral cavity and pharyngeal cancer (aHR, 3.95; 95% CI, 1.21-12.89) and colorectal cancer (aHR, 1.79; 95% CI, 1.12-2.88). Male HS patients had a higher risk of central nervous system cancer (aHR, 3.14; 95% CI, 1.38-7.15), oral cavity and pharyngeal cancer (aHR, 2.80; 95% CI, 1.25-6.25), and prostate cancer (aHR, 2.05; 95% CI, 1.30-3.24). Younger HS patients had higher risks of central nervous system cancer (aHR, 3.05; 95% CI, 1.09-8.56) than younger control patients. Older patients with HS had higher risks of oral cavity and pharyngeal cancer (aHR, 3.26; 95% CI, 1.57-6.80), prostate cancer (aHR, 2.05; 95% CI, 1.30-3.24), and colorectal cancer (aHR, 1.41; 95% CI, 1.04-1.93) than did older control patients.	There is a higher risk of developing oral cavity and pharyngeal cancer, central nervous system cancer, prostate cancer and colorectal cancer in patients with HS.
Bailey et al.; 2023 [13]	Meta-Analysis: 6 included studies (*I*)		Patients with HS had a significantly increased risk of oral and pharyngeal cancers (*p* < 0.001).	There is a significantly risk of developing oral and pharyngeal cancer in patients with HS.

HS, Hidradenitis suppurativa; SIR, standardized incidence ratio; aHR, adjusted hazard ratio; CI, confidence interval.

**Table 6.** Cases reported lymphatic neoplasms in HS patients (*level of evidence: IV*).

Reference (year)	Sex/ Age (years)	Duration of HS prior to lymphoma (years)	HS-Treatment	Hurley stage	Type of lymphoma	Location of lymphoma	Staging	Phenotype	Ki-67	Treatment	Outcome
Calamaro et al. (2016) [40]	M/35	Unknown	excisions	unknown	Intralymphatic proliferation of T-cell lymphoid blasts	Inguinal	Negative	CD4^+^, CD30^+^	>90%	None	Full remission.
Vellaichamy et al. (2020) [41]	F/27	2	adalimumab	III	T-cell/histiocyte-rich large B-cell lymphoma	Liver, retroperitoneal and inguinal lymph nodes	Negative	Unknown	Unknown	Chemotherapy	Full remission.
Preis et al. (2022) [42]	F/56	20	systemic antibiotics	III	Plasmablastic lymphoma	Right axilla	lymphadenopathy in the right submandibular region, bilateral axillae, and the bilateral subpectoral region, positive bone marrow biopsie	CD3^-^	90%	Chemotherapy with cyclophosphamide, doxorubicin, etoposide, vincristine and prednisone and additional bortezomib	Full remission.
Saraiva et al. (2023) [43]	M/72	30	methotrexate and infliximab (*former multiple excisions, systemic antibiotics, percutaneous drainage and fistulotomies*)	unknown	Non-Hodgkin EBV-positive diffuse large B-cell lymphoma	Anal canal and distal rectum	Negative	CD3^-^, CD20^+^	50%	Colostomy and Immunochemotheray with rituximab, cyclophosphamide, doxorubicin, vincristin and prednisolon	Full remission.
Craig et al. (2023) [44]	F/18	0.5	adalimumab	Unknown	EBV-negative, ALK-positive, KRAS-mutant anaplastic large T-cell lymphoma	T12 vertebral and paraspinal	Negative	CD3^-^, CD4^+^, CD20^+^, CD30^+^, CD45^+^	Unknown	brentuximab vedotin	Full remission.

F, female; M, male; HS, Hidradenitis suppurativa.

### 3.5. HS and other malignancies

In addition, Lapins et al. and Jung et al. found a higher incidence of additional malignancies in HS patients compared to the healthy cohort, including lymphoma, NMSC and female genital tract cancers, as well as other malignancies (Table 5: HS and other malignancies) [14,15]. Significant elevations in the relative risk of buccal cancer (SIR, 5.5; 95% CI, 1.8-12.9) and primary hepatocellular carcinoma (SIR, 10.0; 95% CI, 2.1-29.2) were found in the Swedish cohort study conducted by Lapins et al. Conversely, there was an increased risk of oesophageal cancer (SIR, 7.; 95% CI, 2.2-16.6). The study found that there was no significant statistical relevance for lung cancer (SIR, 1.7; 95% CI, 0.6-4.1), kidney cancer (SIR, 1.7; 95% CI, 0.2-6.3), cancers of urinary tract organs other than the kidney (SIR, 1.9; 95% CI, 0.4-5.5) and colorectal carcinoma, breast cancer, malignant genital tumours in women and brain cancer. However, these cancers were associated with comorbidities such as diabetes mellitus, alcohol abuse and nicotine abuse. A significant increase was observed in the hazard ratio for oral cavity and pharyngeal cancer (aHR, 3.10; 95% CI, 1.60-6.02), central nervous system cancer (aHR, 2.40; 95% CI, 1.22-4.70), prostate cancer (aHR, 2.05; 95% CI, 1.30-3.24), and colorectal cancer (aHR, 1.45; 95% CI, 1.09-1.00) [14]. After adjustment for comorbidities, the study by Jung et al. found that in the HS cohort, there was a higher risk for oral cavity and pharyngeal cancer C (aHR, 3.95; 95% CI, 1.21-12.89) and colorectal cancer (aHR, 1.79; 95% CI, 1.12-2.88) in female HS patients. Malignancies of the central nervous system (aHR, 3.14; 95% CI, 1.38-7.15), oral cavity and pharynx (aHR, 2.80; 95% CI, 1.25-6.25), and prostate (aHR, 2.05; 95% CI, 1.30-3.24) had an increased hazard ratio in male HS patients. Younger HS patients (age <40 years) also had an increased risk of central nervous system cancer (aHR, 3.05; 95% CI, 1.09-8.56) and leukaemia (aHR, 2.55; 95% CI, 1.02-6.39) compared with younger controls. Older people diagnosed with HS have an increased risk of developing Hodgkin lymphoma (aHR 9.04; 95% CI, 1.26-64.85), oral and pharyngeal cancer (aHR 3.26; 95% CI, 1.57-6.80), prostate cancer (aHR 2.05; 95% CI, 1.30-3.24) and colorectal cancer (aHR 1.41; 95% CI, 1.04-1.93) compared with older controls [15]. A meta-analysis showed a notable increase in oral and pharyngeal cancers (*p* < 0.001), but no corresponding increase in other cancers [13].

## 4. Discussion

Our systematic review suggests that patients with HS have an increased risk of cancer compared with the general population. A retrospective study by Lapins et al. examined the prevalence of cancer in 2,119 HS patients using review of patient databank, discovering a 50% higher risk of all types of neoplasms compared to standard rates in the Swedish population [14]. In addition, HS patients had a significantly higher incidence of buccal and liver cancer, except for non-melanoma skin cancer. They also discovered that the occurrence of oesophageal, lung, kidney, urinary tract, and hematopoietic cancers increased among HS patients compared to the general population, although this increase was not statistically significant. In this group, up to 88.9% of all subjects were smokers, and 4.1% of HS patients had increased alcohol consumption. These factors may contribute to the higher prevalence of malignancies [14,45]. A remarkable number of patients with HS and cancer had comorbid diabetes mellitus. The reasons for the described connection are currently unclear. Meta-analyses investigating the relationship between cancer and diabetes mellitus have revealed a non-significant correlation, which is most likely due to confounding by comorbidities [46,47]. Jung et al. also observed the risk of cancer in a nationwide population-based study in the Republic of Korea [15]. In that study, after adjusting the HR for comorbidities like dyslipidaemia and hypertension, the overall risk of pharyngeal cancer, Hodgkin lymphoma, central nervous system neoplasms, NMSC, prostate cancer and colorectal cancer was significantly higher in patients with moderate to severe HS than in healthy controls [15]. The authors concluded that patients with HS require heightened cancer surveillance, alongside prioritization of lifestyle modifications, including combating behaviours linked to smoking, alcohol consumption and conditions such as obesity [15]. The results regarding the occurrence of cancer and in particular lymphoma, NMSC and oropharyngeal cancer were also confirmed in a meta-analysis by Bailey et al. [8]. The lack of adjustment for other cancer-promoting risk factors in all three studies constitutes an ascertainment bias.

Several studies have demonstrated a higher incidence of cancer among patients who smoke [14,16,17, 24, 48–52]. The link between nicotine abuse and HS is widely acknowledged [7,45]. Jourabachi et al. suggested that smoking impairs the NOTCH signalling pathway in HS patients [16]. Inhibiting the NOTCH signalling pathway disrupts the homeostasis of hair follicles, leading to follicle rupture and a local inflammatory response [53]. Analysis of mRNA and protein expression shows increased levels of components of the NOTCH signalling pathway in HS [54]. In terms of cancer, NOTCH is known to both facilitate and inhibit carcinogenesis [55]. NOTCH components have been shown to have an oncogenic function and be overexpressed in various types of cancer, including acute and chronic lymphocytic leukaemia’s [56–59], colorectal carcinomas [59–61], gliomas [62], and prostate carcinomas [63]. These carcinomas occur more frequently in HS patients [13–15]. In SCC, NOTCH has a tumour-suppressive effect [53], but mutations in NOTCH receptors have been observed in cSCC, resulting in their dysfunction or downregulation [64]. Mutations were also detected in both HPV-positive and HPV-negative tumours in vulvovaginal SCCs. Loss of function was demonstrated for NOTCH-1 and tumour suppressor gene 53 (TP53) mutations, particularly in HPV-independent processes [65]. In head and neck tumours, Mutations in NOTCH-1 indicate a function as a tumour suppressor, while activation of the NOTCH signalling pathway suggests a proto-oncogenic effect [66]. Overexpression of NOTCH components in HS may escalate mutation events and increase the risk of carcinogenic events, especially for in carcinogenesis of cSCC arising from HS.

**Figure 3.**
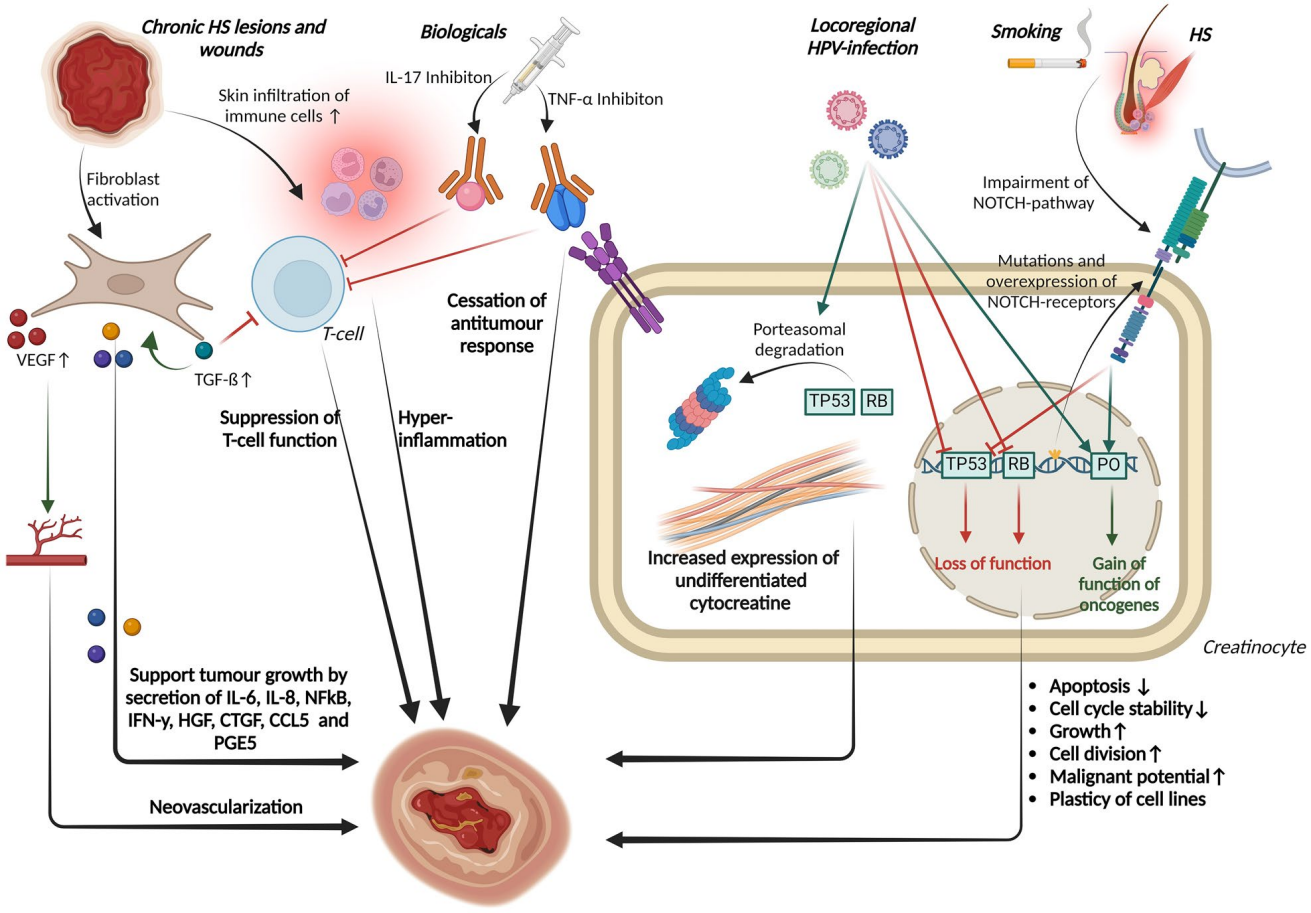
Shows an overview of possible pathomechanisms of malignant transformation of chronic Hidradenitis suppurativa (HS) into cutaneous squamous cell carcinoma or marjolin ulcer. Genetic alterations in Hidradenitis suppurativa (HS) and comorbid smoking lead to impairment of the NOTCH signaling pathway, which activates the expression of proto-oncogenes (POs) and suppresses that of the tumour suppressor gene 53 (TP53). Locoregional infections with high-risk human papillomaviruses (HPV) support the genetic effects of malignant development. Increased expression of undifferentiated cytocreatins can be observed in creatinocytes. Treatment with biologics can impair the anti-tumour response of T cells and other body cells. Chronic wounds can promote degeneration through recruitment and activation of fibroblasts, deposition of extracellular matrix components, infiltration of immune cells and hyperinflammation, neovascularisation and cell lineage plasticity. RB, Retinoblastom-Protein; HGF, hepatocyte growth factor; CTGF, connective growth factor.

The reviewed articles provide specific data regarding the occurrence of cSCC, in lesions caused by long-standing HS. The estimated prevalence of this complication is between 0.5% and 4.6% [24]. It is notable that even though HS is more common in women than in men, all but one study reported a higher incidence of cSCC in men [4,24,47]. Several studies have shown a higher incidence of cSCC in the buttock, gluteal, and anogenital regions affected by HS [16,28,29,48–52,67,68]. No cases have been reported in the axillary region to date. It is noteworthy that the age of onset was lower in HS patients than in non-HS patients, regardless of gender [14,15]. In addition, the rates of both lymph node and distant metastases in cSCC are higher in HS patients than in the general population [17,69], leading to a significantly reduced survival rate [17]. These results were confirmed in case reports and reviews [16,48–52,67,68]. Furthermore, therapy involving further excision of the affected areas and immunotherapy or a combination of both resulted in better treatment outcomes [16,48–52,67,68]. In contrast, treatment with Mohs micrographic surgery was associated with an increased mortality rate [16,28, 48–52,67,68]. These findings highlight the high malignant potential of cSCC arising from HS and underscore the importance of early, hard intervention. Important risk factors for cSCC include chronic sun exposure, aging, having light-coloured skin, and immunosuppression. Additionally, cSCC has been observed in cases of persistent wounds or chronic inflammation [69–72]. Regardless of the underlying cause, a mutation in TP53 is always present [69,73]. The pathogenesis of cSCC in chronic inflammation differs from that under chronic sun exposure, although the exact mechanisms remain unknown. Figure 3 shows an overview of possible pathomechanisms of malignant transformation of chronic Hidradenitis suppurativa (HS) into cSCC or Marjolin ulcer. Kurakowa et al. observed an infundibular-like keratinized epithelium (type A) in different cases of cSCC arising from HS [74]. The histological examinations support the thesis of a malignant transformation of the type A epithelium. This is indicated by an increase in undifferentiated creatinine (CK14), the expression of simple epithelial keratins (CK 7, 8, 18 and 19) and a downregulation of stratified and differentiated creatinines (CK 1 and 10) [74]. Fabbrocini et al. proposed that a “immunocompromised cutaneous area” with impaired immune control could explain malignant transformation or infections through dysimmune responses [75]. In HS, immune dysregulation can be observed in the affected areas of the body. Scarring leads to disruption of lymphatic microcirculation and peripheral nerve endings, impairing the interaction between lymphocyte-derived immune cells and peripheral nerve neurotransmitters. Destabilization of this relationship may promote tumours. Conversely, excessive interaction between immune cells and nerve endings leads to immune dysfunction and response in localized scar areas, making it more difficult for the immune system to recognize tumour cells. Lymphoedema, which is a common complication of severe HS, can therefore impede the flow of immunogenic cells to affected regions, increasing the risk of malignant degeneration [76,77].

Genital HPV infection is more common in HS patients [78]. Oncogenic HPV16 DNA was detected growing cSCC on HS in over 30% of all cases in the anal, perianal, buttock, thigh and groin regions. It can be concluded that HPV16 is involved in the degeneration of these regions [16,27]. The development of both mucosal and cutaneous SCC is known to be significantly influenced by HPV [56,57]. This is due to the integration of the viral genome into the infected epithelial cells, which facilitates continuous viral DNA replication [79]. Binding to the retinoblastoma protein (Rb), the synthesized HPV E7 protein promotes its expression and ubiquitination while the viral E6 protein targets proteosome degradation of TP53. This leads to inhibition of apoptosis and blocking of cell cycle checkpoints, resulting in atypical cell proliferation and dysplasia development [80]. Malignancies can develop later due to these changes [80]. Scheinfield et al. suggest a synergistic effect between chronic inflammation, cellular immune response impairment, and HPV infection that promotes malignancy development [81]. Flores et al. found a significant correlation indicating autoinoculation of HPV-16 viral load in proximal anatomical sites of the male anogenital region. This correlation is favoured by poor hygiene and humidity in male HPV-positive HS patients [77,82]. The hypothesis that HPV is involved in the malignant degeneration of HS could also account for the higher incidence of carcinomas in the female genitalia of patients with severe HS [30–38]. The two primary HPV genotypes responsible for vulvar cancer and its precursor lesions (VIN I-III) are HPV16 and HPV33 [83]. High-risk HPV16 infections have become more prevalent in cases of vulvar carcinoma among HS patients [37]. Additionally, HPV infections are linked to SCC of the mouth and pharynx [79,84,85]. Population-based studies, as well as a meta-analysis, have indicated a significantly higher risk of these carcinomas among HS patients [13,15]. According to Jung et al. male HS patients have a heightened incidence of prostate carcinomas, a type of tumour that can also result from frequent infections with high-risk HPV [15,86]. Consequently, it is plausible that HPV high-risk types, particularly HPV16, contribute to the development of SCCs in HS patients. It is likely that lesions in the anogenital region of HS patients may have an increased potential for degeneration due to local HPV infection. As a result, preventive HPV vaccination could be beneficial for this group [17,67].

In certain HS patients, the use of TNF-α inhibitors heightens the probability of cSCC onset while decreasing the time it takes to develop [16,37,38,48,67]. Cases of lymphoma have also been reported in HS patients receiving systemic therapy with TNF-α inhibitors. As per the guidelines, patients with moderate-to-severe HS, in particular the patient group with the greatest complication rate, can be treated with those biologicals [87,88]. The association between the use of TNF-α inhibitors and cancer development has been debated. Some studies have reported an increased risk of certain cancers such as NHL, HL and NMSC in users of TNF-α inhibitors [89,90]. However, the association between TNF-α inhibitors and cancer has not been replicated in other studies [91,92]. The role of TNF- α antagonists in causing NMSC and lymphomas has not yet been defined. TNF-α, a major cytokine in the tumour microenvironment, may promote the progression of tumours [93], while inhibiting the cytokine may cause a cessation of the antitumour immune response, thereby promoting the growth of immunogenic tumours [94]. TNF-α also plays a role in the killing of tumour cells by natural killer cells and CD8+ lymphocytes [95,96]. TNF-α receptors appear to be involved in tumour surveillance [97]. Based on a meta-analysis of observational studies on skin cancer incidence in biologic-treated psoriasis, psoriatic arthritis, and HS patients, there was no increase in the incidence of NMSC and melanoma when compared to non-biologic treatment [94]. No studies involving HS patients were included in the final analysis due to a lack of evidence. However, confounding by light therapy and DMARD immunosuppressants cannot be excluded in psoriasis patients [94]. In addition, an increase in the risk of NMSC, particularly cSCC, and melanoma was observed in further meta-analyses with patient groups with previous rheumatic disease or psoriasis [89,98–101]. Some studies have shown an increased risk of lymphoma in rheumatoid arthritis patients with TNF-α inhibition [102,103]. These findings have not been confirmed in other studies [104,105]. In a population-based study of cancer risk under TNF-α inhibition in psoriasis patients by Jung et al. a significant increase in the risk of lymphoma in general and NHL was demonstrated [106]. In case reports on HS patients who developed lymphoma under TNF- α inhibition, only the occurrence of NHL, particularly two cases of B-cell lymphoma, was reported [41,43,44]. Therefore, a confounding aspect in the development of lymphoma in anti TNF-α treated HS can be discussed. In contrast, comparative pharmacovigilance studies did not show an increased risk of lymphoma under treatment with adalimumab and infliximab [107]. An increase in the general risk of cancer was also not observed with TNF-α inhibition [89, 98–101]. In conclusion, the conflicting results highlight the need for further studies to validate the role of TNF-α inhibitors in tumour development. IL-17 inhibitors are other biologics that have been approved for the treatment of moderate-to-severe HS and used for a while in psoriasis therapy [108,109]. A meta-analysis by Bilal et al. showed an increased risk of cancer in rheumatic patients treated with IL inhibitors [110]. As IL-17 inhibitors were only recently introduced for the treatment of HS and no cases of cancer were reported to date, it is not yet possible to say whether these results can be extrapolated to HS patients. In conclusion, the use of biologics in the treatment of HS can at least be considered a confounding factor for the development of malignancies.

In addition to cSCC, several studies have documented the presence of Marjolin’s ulcers. These are rare but severe degeneration of scars, long-standing wounds and ulcers, and persistent inflammation, particularly SCC [21]. There is a deficiency of prospective information currently accessible, hence the authors gauge the occurrence rate of Marjolin ulcer in HS to be between 2.78% and 4.1% [111,112]. Marjolin ulcers are rare, more prevalent in males than in females and characterized by aggressive growth, rapid metastatic tendency, high recurrence rate and poorer prognosis than other NMSC [21–23,113]. Beard et al. propose a multifactorial pathogenesis of Marjolin ulcers resulting from chronic irritation, ulceration, and increased tissue temperature. This process weakens the epithelium, increasing susceptibility to carcinogens [114]. Similarities between tumour growth and wound healing include the recruitment and activation of fibroblasts, the deposition of extracellular matrix components, the infiltration of immune cells, neovascularisation and the plasticity of cell lines. Fibroblasts mediate the expression of α-smooth-muscle actin in both wound healing and neoplasm growth [115–118]. Fibroblasts contribute to oncogenesis by influencing the tumour microenvironment through paracrine signalling, regulation of the immune response, and incorporation of extracellular matrix components. They also stimulate neoangiogenesis and increase TGF-ß secretion *via* positive feedback, thereby promoting further fibroblast growth and suppressing T-cell function. Fibroblast clones secrete IL-6, IL-8, NFkB, IFN-y, HGF, CTGF, CCL5, and PGE5 to promote tumour growth. Additionally, neovascularisation is stimulated by secretion of VGEF [119]. In HS patients have been found to have increased serum levels of IL-6, IL-8 and VGEF compared to healthy subjects [120–125]. Furthermore, dysregulation of cadherin-mediated cell-cell adhesions has been demonstrated in HS and Marjolin ulcers [125,126]. It can be assumed that increased inflammatory activity, a stroma rich in growth factors and predominant neovascularisation are involved in the development of Marjolin ulcers in HS lesions.

The burden of comorbidities in HS patients was investigated in a retrospective case-control study by Shylankievch et al. Among other findings, the study demonstrated an elevated incidence of lymphoma in the HS cohort [24]. The results were further substantiated by Tannenbaum et al. [39]. In this cross-sectional cohort analysis, the authors evaluated the prevalence of NHL, HL, and CTLC in HS patients. The results showed that HS patients have a 2 to 4fold increased risk of developing lymphoma compared to the general population. The researchers postulate a clonal progression of immune cell populations owing to persistent inflammatory activity [39]. In addition, Jung et al. found that young HS patients have an increased risk of developing leukaemia, while older patients have a significantly increased risk of NHL [15]. Unlike other malignancies, case studies suggest that lymphomas arise irrespective of the duration of the illness or the patient’s age [40–44]. It should be noted that the exact pathogenetic correlations between HS and lymphoma are currently not established [41]. Increased expression of IL-17 and IL-23 as well as Th17 is involved in the pathogenesis of HS [127]. In addition, the expression of IL-36 is increased in HS lesions [128]. IL-17 and −23 have also been detected in increased concentrations in NK/T lymphomas [129,130]. Moreover, specific TNF-α haplotypes were observed to promote the progression of B-cell lymphomas [131]. These findings support the hypothesis that increased immune activation and accelerated inflammatory cell turnover may explain the increased incidence of lymphoma in chronic inflammatory skin diseases [39].

Over-activation of the immune system could also be another reason for the increased incidence of solid tumours in HS patients. In support of this hypothesis, meta-analyses have shown a significant increase in cancer risk for elevated IL-17 levels, as well as for colorectal and oral cancers [132]. An association with hepatocellular carcinoma (HCC) has been shown for common IL-23 polymorphisms [133]. An involvement of IL-36 in HCC and colorectal cancer and their prognosis has also been demonstrated [134].

These explanatory approaches are supported at the molecular level by a chronic subclinical proinflammatory milieu in both obesity and HS, which is characterised by a comparable cytokine profile [135–137]. Obesity, a disease associated with HS, can shift the body’s homeostasis in a direction that favours macrophage invasion and polarisation. M1-type macrophages then release increased levels of cytokines, including TNF-α, IL-1, IL-6, IL-12 and IL-23, oxygen free radicals and nitric oxide (NO), as well as free RNAs. In addition to hyperinflammation of the HS, these induced proinflammatory processes also support inflammatory and tissue-destructive processes in various tissues [135]. It is therefore possible that meta-inflammation in obese HS patients may contribute to the development of malignancy in this patient group.

No predictive models exist for which HS patients are most likely to develop cancer. As mentioned above, lifestyle is an important factor [15]. This is particularly relevant for nicotine consumption and metabolic syndrome [14,16,17,24,48–52]. Also, a longer disease duration seems to be associated with an increased incidence of malignant tumours [14,15,24]. The pan-immune-inflammation-value (PIV) is a biomarker whose levels correlate with the progression of HS disease, as well as with the occurrence, treatment success and survival of malignancies [138,139]. This could be a possible approach for prediction. In general, the combination of regular monitoring of HS, analysis of individual risk factors, continuous cancer prevention and participation in recommended cancer screening should be consistently recommended to HS patients in order to identify patients at high risk of developing cancer as early as possible.

### 4.1. Strengths and limitations

This systematic literature review comprises 25 articles, which makes it the largest of its kind to date in the field of time course studies of HS and malignancies. Our systematic approach, which adheres to the PRISMA guidelines, ensures that all relevant studies have been included. However, we limited our review to articles in English or German that were freely accessible. As a result, many potentially valuable articles that were not freely accessible could not be included. In comparison to previously published reviews on this topic, our work represents a significant strength, as it did not include reviews, abstracts, letters to the editor, or case reports. Furthermore, a critical appraisal of the included studies was carried out using the GRADE scale to secure the quality of this review.

A potential limitation of including meta-analyses in this systematic review is that the same studies are included in both this and the present review. Consequently, we elected to derive conclusions solely from statistical outcomes and the pivotal messages of the meta-analyses. Nevertheless, the incorporation of meta-analyses into systematic reviews facilitates a highly substantiated and more comprehensive assessment of the subject matter.

A major limitation of the studies analysed is that comorbidities, which can increase the risk of malignant disease, were frequently not considered. This introduces the possibility of bias in the results. Only Jung et al. adjusted the hazard ratio for comorbid dyslipidaemia and hypertension [15]. Moreover, most of the studies included in this review were conducted in highly economically developed countries, which could limit the generalisability of the data found. Notwithstanding these two limitations, the majority of the data presented were replicated in at least one other study.

## 5. Conclusion

In summary, studies have shown that patients with HS have an increased incidence of malignancies, particularly cSCC and lymphoma. The cSCC in HS is a multifactorial malignancy arising from chronically inflamed regions. However, there is still a lack of experimental research investigating the role of chronic inflammation such as HS in carcinogenesis. Further research in this area could provide a valuable basis for promising future studies. Other cancers, including oropharyngeal, CNS, colorectal and prostate cancers, have also been associated with HS. HS patients should be informed of the increased risk of cancer.

## Data Availability

The data that support the findings of this study are available from the corresponding author, [NA], upon reasonable request.

## Registration and protocol

This review has not been registered. No protocol was created.

## References

[1] Kirschke J, Hessam S, Bechara FG. Hidradenitis suppurativa/Acne inversa: ein Update [hidradenitis suppurativa/acne inversa: an update]. Hautarzt. 2015;66(6):413–422. doi: 10.1007/s00105-015-3616-y. 25877488

[2] Jfri A, Nassim D, O’Brien E, et al. Prevalence of hidradenitis suppurativa: a systematic review and meta-regression analysis. JAMA Dermatol. 2021;157(8):924–931. doi: 10.1001/jamadermatol.2021.1677. 34037678 PMC8156162

[3] Ingram JR, Collins H, Atkinson MD, et al. The prevalence of hidradenitis suppurativa is shown by the Secure Anonymised Information Linkage (SAIL) Databank to be one per cent of the population of Wales. Br J Dermatol. 2020;183(5):950–952. doi: 10.1111/bjd.19210. 32433788

[4] Ingram JR. The epidemiology of hidradenitis suppurativa. Br J Dermatol. 2020;183(6):990–998. doi: 10.1111/bjd.19435. 32880911

[5] Lee EY, Alhusayen R, Lansang P, et al. What is hidradenitis suppurativa? Can Fam Physician. 2017;63(2):114–120. 28209676 PMC5395382

[6] Zouboulis CC, Bechara FG, Fritz K, et al. S2k-Leitlinie zur Therapie der Hidradenitis suppurativa/Acne inversa (ICD-10-Code: l 73.2). Aktuelle Dermatologie. 2024;50(01/02):30–83. doi: 10.1055/a-2225-7983.

[7] Nguyen TV, Damiani G, Orenstein LAV, et al. Hidradenitis suppurativa: an update on epidemiology, phenotypes, diagnosis, pathogenesis, comorbidities and quality of life. J Eur Acad Dermatol Venereol. 2021;35(1):50–61. doi: 10.1111/jdv.16677. 32460374

[8] Abu Rached N, Gambichler T, Ocker L, et al. Screening for diabetes mellitus in patients with hidradenitis suppurativa-a monocentric study in Germany. Int J Mol Sci. 2023;24(7):6596. doi: 10.3390/ijms24076596. 37047569 PMC10094965

[9] Goldburg SR, Strober BE, Payette MJ. Hidradenitis suppurativa: epidemiology, clinical presentation, and pathogenesis. J Am Acad Dermatol. 2020;82(5):1045–1058. doi: 10.1016/j.jaad.2019.08.090. 31604104

[10] Phan K, Tatian A, Woods J, et al. Prevalence of inflammatory bowel disease (IBD) in hidradenitis suppurativa (HS): systematic review and adjusted meta-analysis. Int J Dermatol. 2020;59(2):221–228. doi: 10.1111/ijd.14697. 31631340

[11] Almuhanna N, Finstad A, Alhusayen R. Association between hidradenitis suppurativa and inflammatory arthritis: a systematic review and meta-analysis. Dermatology. 2021;237(5):740–747. doi: 10.1159/000514582. 33774640

[12] Phan K, Huo YR, Smith SD. Hidradenitis suppurativa and psychiatric comorbidities, suicides and substance abuse: systematic review and meta-analysis. Ann Transl Med. 2020;8(13):821–821. doi: 10.21037/atm-20-1028. 32793666 PMC7396254

[13] Bailey AMJ, Li HO, Tan MG, et al. Association of hidradenitis suppurativa and malignancy: a systematic review and meta-analysis. J Eur Acad Dermatol Venereol. 2023;37(9):e1107–e1108. doi: 10.1111/jdv.19136. 37143377

[14] Lapins J, Ye W, Nyrén O, et al. Incidence of cancer among patients with hidradenitis suppurativa. Arch Dermatol. 2001;137(6):730–734. 11405761

[15] Jung JM, Lee KH, Kim Y, et al. Assessment of overall and specific cancer risks in patients with hidradenitis suppurativa. JAMA Dermatol. 2020;156(8):844–853. doi: 10.1001/jamadermatol.2020.1422. 32459291 PMC7254443

[16] Jourabchi N, Fischer AH, Cimino-Mathews A, et al. Squamous cell carcinoma complicating a chronic lesion of hidradenitis suppurativa: a case report and review of the literature. Int Wound J. 2017;14(2):435–438. doi: 10.1111/iwj.12671. 27681476 PMC7949826

[17] Racanelli E, Jfri A, Gefri A, et al. Cutaneous squamous cell carcinoma in patients with hidradenitis suppurativa. Cancers (Basel). 2021;13(5):1153. doi: 10.3390/cancers13051153. 33800250 PMC7962537

[18] Dreyfuss I, Kamath P, Frech F, et al. Squamous cell carcinoma: 2021 updated review of treatment. Dermatol Ther. 2022;35(4):e15308. doi: 10.1111/dth.15308. 34997811

[19] Guyatt GH, Oxman AD, Vist GE, GRADE Working Group, et al. GRADE: an emerging consensus on rating quality of evidence and strength of recommendations. BMJ. 2008;336(7650):924–926. doi: 10.1136/bmj.39489.470347.AD. 18436948 PMC2335261

[20] Bazaliński D, Przybek-Mita J, Barańska B, et al. Marjolin’s ulcer in chronic wounds - review of available literature. Contemp Oncol (Pozn). 2017;21(3):197–202. doi: 10.5114/wo.2017.70109. 29180925 PMC5701580

[21] Shah M, Crane JS, Marjolin U. StatPearls. Treasure Island: StatPearls; 2023.

[22] Sadegh Fazeli M, Lebaschi AH, Hajirostam M, et al. Marjolin’s ulcer: clinical and pathologic features of 83 cases and review of literature. Med J Islam Repub Iran. 2013;27(4):215–224. 24926183 PMC4011412

[23] Giesey R, Delost GR, Honaker J, et al. Metastatic squamous cell carcinoma in a patient treated with adalimumab for hidradenitis suppurativa. JAAD Case Rep. 2017;3(6):489–491. doi: 10.1016/j.jdcr.2017.08.017. 29022006 PMC5633337

[24] Shlyankevich J, Chen AJ, Kim GE, et al. Hidradenitis suppurativa is a systemic disease with substantial comorbidity burden: a chart-verified case-control analysis. J Am Acad Dermatol. 2014;71(6):1144–1150. doi: 10.1016/j.jaad.2014.09.012. 25440440

[25] Brown ID, Adawi W, Saal R, et al. Associations between hidradenitis suppurativa and dermatological conditions in adults: a national cross-sectional study. Clin Exp Dermatol. 2023;48(5):524–527. doi: 10.1093/ced/llad034. 36702792

[26] Ritz JP, Runkel N, Haier J, et al. Extent of surgery and recurrence rate of hidradenitis suppurativa. Int J Colorectal Dis. 1998;13(4):164–168. doi: 10.1007/s003840050159. 9810520

[27] Lavogiez C, Delaporte E, Darras-Vercambre S, et al. Clinicopathological study of 13 cases of squamous cell carcinoma complicating hidradenitis suppurativa. Dermatology. 2010;220(2):147–153. doi: 10.1159/000269836. 20029163

[28] Matoso A, Ross HM, Chen S, et al. Squamous neoplasia of the scrotum: a series of 29 cases. Am J Surg Pathol. 2014;38(7):973–981. doi: 10.1097/PAS.0000000000000192. 24618607

[29] Kohorst JJ, Shah KK, Hallemeier CL, et al. Squamous cell carcinoma in perineal, perianal, and gluteal hidradenitis suppurativa: experience in 12 patients. Dermatol Surg. 2019;45(4):519–526. doi: 10.1097/DSS.0000000000001713. 30608295 PMC6449199

[30] Manolitsas T, Biankin S, Jaworski R, et al. Vulval squamous cell carcinoma arising in chronic hidradenitis suppurativa. Gynecol Oncol. 1999;75(2):285–288. doi: 10.1006/gyno.1999.5547. 10525388

[31] Crain VA, Gulati S, Bhat S, et al. Marjolin’s ulcer in chronic hidradenitis suppurativa. Am Fam Physician. 2005;71(9):1652, 1657. 15887445

[32] Short KA, Kalu G, Mortimer PS, et al. Vulval squamous cell carcinoma arising in chronic hidradenitis suppurativa. Clin Exp Dermatol. 2005;30(5):481–483. doi: 10.1111/j.1365-2230.2005.01875.x. 16045671

[33] Maclean GM, Coleman DJ. Three fatal cases of squamous cell carcinoma arising in chronic perineal hidradenitis suppurativa. Ann R Coll Surg Engl. 2007;89(7):709–712. doi: 10.1308/003588407X209392. 17959012 PMC2121284

[34] Pena ZG, Sivamani RK, Konia TH, et al. Squamous cell carcinoma in the setting of chronic hidradenitis suppurativa; report of a patient and update of the literature. Dermatol Online J. 2015;21(4). 25933081

[35] Rekawek P, Mehta S, Andikyan V, et al. Squamous cell carcinoma of the vulva arising in the setting of chronic hidradenitis suppurativa: a case report. Gynecol Oncol Rep. 2016;16:28–30. doi: 10.1016/j.gore.2016.03.002. 27331133 PMC4899419

[36] Makris GM, Poulakaki N, Papanota AM, et al. Vulvar, perianal and perineal cancer after hidradenitis suppurativa: a systematic review and pooled analysis. Dermatol Surg. 2017;43(1):107–115. doi: 10.1097/DSS.0000000000000944. 27755170

[37] Bessaleli E, Scheinfeld N, Kroumpouzos G. Squamous cell carcinoma of the cervix arising in a patient on adalimumab – a need for cervical screenings in patients on tumor necrosis factor inhibitors. Dermatol Online J. 2018;24. 30142745

[38] Vergeldt TFM, Driessen RJB, Bulten J, et al. Vulvar cancer in hidradenitis suppurativa. Gynecol Oncol Rep. 2022;39:100929. doi: 10.1016/j.gore.2022.100929. 35106355 PMC8784320

[39] Tannenbaum R, Strunk A, Garg A. Association between hidradenitis suppurativa and lymphoma. JAMA Dermatol. 2019;155(5):624–625. doi: 10.1001/jamadermatol.2018.5230. 30698627 PMC6506877

[40] Calamaro P, Cerroni L. Intralymphatic proliferation of T-cell lymphoid blasts in the setting of hidradenitis suppurativa. Am J Dermatopathol. 2016;38(7):536–540. doi: 10.1097/DAD.0000000000000466. 26844617

[41] Vellaichamy G, Townsend SM, Lyons AB, et al. T-cell/histiocyte-rich large B-cell lymphoma in a 27-year-old with hidradenitis suppurativa, psoriasis, and vitiligo: implications for screening. JAAD Case Rep. 2020;6(12):1252–1253. doi: 10.1016/j.jdcr.2020.08.032. 33294556 PMC7700968

[42] Preis S, Kain A, Biedermann T, et al. Plasmablastic lymphoma masked by hidradenitis suppurativa. JAAD Case Rep. 2022;27:13–15. doi: 10.1016/j.jdcr.2022.06.016. 35990238 PMC9389130

[43] Saraiva RO, Saunders C, Varela dos Santos M, et al. Refractory hidradenitis suppurativa: a diagnosis to consider. GE Port J Gastroenterol. 2023;31(1):60–64. doi: 10.1159/000528432. 38476303 PMC10928865

[44] Craig A, Wen KW. ALK + ALCL in the setting of adalimumab-related hidradenitis suppurativa. Clin Case Rep. 2023;11(4):e7156. 37064740 10.1002/ccr3.7156 PMC10090933

[45] König A, Lehmann C, Rompel R, et al. Cigarette smoking as a triggering factor of hidradenitis suppurativa. Dermatology. 1999;198(3):261–264. doi: 10.1159/000018126. 10393449

[46] Tsilidis KK, Kasimis JC, Lopez DS, et al. Type 2 diabetes and cancer: umbrella review of meta-analyses of observational studies. BMJ. 2015;350(jan02 1):g7607. doi: 10.1136/bmj.g7607. 25555821

[47] Wang Y, Yan P, Fu T, et al. The association between gestational diabetes mellitus and cancer in women: a systematic review and meta-analysis of observational studies. Diabetes Metab. 2020;46(6):461–471. doi: 10.1016/j.diabet.2020.02.003. 32097717

[48] Chapman S, Delgadillo D, Barber C, et al. Cutaneous squamous cell carcinoma complicating hidradenitis suppurativa: a review of the prevalence, pathogenesis, and treatment of this dreaded complication. Acta Dermatovenerologica Alpina, Pannonica Adriat. 2018;27:25–28. 29589641

[49] Nielsen VW, Jørgensen AR, Thomsen SF. Fatal outcome of malignant transformation of hidradenitis suppurativa: a case report and literature review. Clin Case Rep. 2020;8(3):504–507. doi: 10.1002/ccr3.2608. 32185045 PMC7069843

[50] Lee SJ, Lim JM, Lee SH, et al. Invasive cutaneous squamous cell carcinoma arising from chronic hidradenitis suppurativa: a case report of treatment by slow mohs micrographic surgery. Ann Dermatol. 2021;33(1):68–72. doi: 10.5021/ad.2021.33.1.68. 33911814 PMC7875211

[51] Atri S, Ben Mahmoud A, Zehani A, et al. The management of hidradenitis suppurativa degenerating into squamous cell carcinoma: about three case reports. Ann Med Surg (Lond). 2021;64:102239. doi: 10.1016/j.amsu.2021.102239. 33868679 PMC8040103

[52] Lahham EE, Billan S, Atrash F, et al. A late presentation of inguinoscrotal cutaneous squamous cell carcinoma (cSCC) masquerading as hidradenitis suppurativa-a case report. J Surg Case Rep. 2023;2023(8):rjad459. 37564053 10.1093/jscr/rjad459 PMC10411990

[53] Li X, Jiang L, Huang Y, et al. A gene dysfunction module reveals the underlying pathogenesis of hidradenitis suppurativa: an update. Austr J Dermatol. 2020;61:e10–e14. 10.1111/ajd.13107 31266094

[54] Hessam S, Gambichler T, Skrygan M, et al. Increased expression profile of NCSTN, Notch and PI3K/AKT3 in hidradenitis suppurativa. J Eur Acad Dermatol Venereol. 2021;35(1):203–210. doi: 10.1111/jdv.16962. 32978818

[55] Zhou B, Lin W, Long Y, et al. Notch signaling pathway: architecture, disease, and therapeutics. Signal Transduct Target Ther. 2022;7(1):95. doi: 10.1038/s41392-022-00934-y. 35332121 PMC8948217

[56] Weng AP, Ferrando AA, Lee W, et al. Activating mutations of NOTCH1 in human T cell acute lymphoblastic leukemia. Science. 2004;306(5694):269–271. doi: 10.1126/science.1102160. 15472075

[57] Bernasconi-Elias P, Hu T, Jenkins D, et al. Characterization of activating mutations of NOTCH3 in T-cell acute lymphoblastic leukemia and anti-leukemic activity of NOTCH3 inhibitory antibodies. Oncogene. 2016;35(47):6077–6086. doi: 10.1038/onc.2016.133. 27157619 PMC5102827

[58] Rosati E, Sabatini R, Rampino G, et al. Constitutively activated Notch signaling is involved in survival and apoptosis resistance of B-CLL cells. Blood. 2009;113(4):856–865. doi: 10.1182/blood-2008-02-139725. 18796623

[59] Tardivon D, Antoszewski M, Zangger N, et al. Notch signaling promotes disease initiation and progression in murine chronic lymphocytic leukemia. Blood. 2021;137(22):3079–3092. doi: 10.1182/blood.2020006701. 33512383

[60] Fre S, Pallavi SK, Huyghe M, et al. Notch and Wnt signals cooperatively control cell proliferation and tumorigenesis in the intestine. Proc Natl Acad Sci U S A. 2009;106(15):6309–6314. doi: 10.1073/pnas.0900427106. 19251639 PMC2649205

[61] Jackstadt R, van Hooff SR, Leach JD, et al. Epithelial NOTCH signaling rewires the tumor microenvironment of colorectal cancer to drive poor-prognosis subtypes and metastasis. Cancer Cell. 2019;36(3):319–336.e7. doi: 10.1016/j.ccell.2019.08.003. 31526760 PMC6853173

[62] Natarajan S, Li Y, Miller EE, et al. Notch1-induced brain tumor models the sonic hedgehog subgroup of human medulloblastoma. Cancer Res. 2013;73(17):5381–5390. doi: 10.1158/0008-5472.CAN-13-0033. 23852537 PMC3766480

[63] Bertrand FE, McCubrey JA, Angus CW, et al. NOTCH and PTEN in prostate cancer. Adv Biol Regul. 2014;56:51–65. doi: 10.1016/j.jbior.2014.05.002. 24933481

[64] South AP, Purdie KJ, Watt SA, et al. NOTCH1 mutations occur early during cutaneous squamous cell carcinogenesis. J Invest Dermatol. 2014;134(10):2630–2638. doi: 10.1038/jid.2014.154. 24662767 PMC4753672

[65] Salama AM, Momeni-Boroujeni A, Vanderbilt C, et al. Molecular landscape of vulvovaginal squamous cell carcinoma: new insights into molecular mechanisms of HPV-associated and HPV-independent squamous cell carcinoma. Mod Pathol. 2022;35(2):274–282. doi: 10.1038/s41379-021-00942-3. 34650187 PMC9450957

[66] Fukusumi T, Califano JA. The NOTCH pathway in head and neck squamous cell carcinoma. J Dent Res. 2018;97(6):645–653. doi: 10.1177/0022034518760297. 29489439 PMC5960881

[67] Roy CF, Roy SF, Ghazawi FM, et al. Cutaneous squamous cell carcinoma arising in hidradenitis suppurativa: a case report. SAGE Open Med Case Rep. 2019;7:2050313X19847359. doi: 10.1177/2050313X19847359. PMC6537059 31205707

[68] Ruggiero A, Lauro W, Miano C, et al. Advanced squamous cell carcinoma developed on chronic hidradenitis suppurativa, successfully treated with cemiplimab: a case report. Case Rep Dermatol. 2023;15(1):35–39. doi: 10.1159/000525347. 36817850 PMC9929653

[69] Thompson AK, Kelley BF, Prokop LJ, et al. Risk factors for cutaneous squamous cell carcinoma recurrence, metastasis, and disease-specific death: a systematic review and meta-analysis. JAMA Dermatol. 2016;152(4):419–428. doi: 10.1001/jamadermatol.2015.4994. 26762219 PMC4833641

[70] Novick M, Gard DA, Hardy SB, et al. Burn scar carcinoma: a review and analysis of 46 cases. J Trauma. 1977;17(10):809–817. doi: 10.1097/00005373-197710000-00010. 909123

[71] Knackstedt TJ, Collins LK, Li Z, et al. Squamous cell carcinoma arising in hypertrophic lichen planus: a review and analysis of 38 cases. Dermatol Surg. 2015;41(12):1411–1418. doi: 10.1097/DSS.0000000000000565. 26551772

[72] B Xiang F, Lucas R, Hales S, et al. Incidence of nonmelanoma skin cancer in relation to ambient UV radiation in white populations, 1978-2012: empirical relationships. JAMA Dermatol. 2014;150(10):1063–1071. doi: 10.1001/jamadermatol.2014.762. 25103031

[73] Wikonkal NM, Brash DE. Ultraviolet radiation induced signature mutations in photocarcinogenesis. J Investig Dermatol Symp Proc. 1999;4(1):6–10. doi: 10.1038/sj.jidsp.5640173. 10537000

[74] Kurokawa I, Nishimura K, Yamanaka K, et al. Cytokeratin expression in squamous cell carcinoma arising from hidradenitis suppurativa (acne inversa). J Cutan Pathol. 2007;34(9):675–678. doi: 10.1111/j.1600-0560.2006.00680.x. 17696913

[75] Fabbrocini G, Ruocco E, De Vita V, et al. Squamous cell carcinoma arising in long-standing hidradenitis suppurativa: an overlooked facet of the immunocompromised district. Clin Dermatol. 2017;35(2):225–227. doi: 10.1016/j.clindermatol.2016.10.019. 28274364

[76] Chu EY, Kovarik CL, Lee RA. Lymphedematous verrucous changes simulating squamous cell carcinoma in long-standing hidradenitis suppurativa. Int J Dermatol. 2013;52(7):808–812. doi: 10.1111/j.1365-4632.2011.05361.x. 22640236

[77] Li Pomi F, Macca L, Motolese A, et al. Neoplastic implications in patients suffering from hidradenitis suppurativa under systemic treatments. Biomedicines. 2021;9(11):1594. doi: 10.3390/biomedicines9111594. 34829823 PMC8615387

[78] Jemec GB, Heidenheim M, Nielsen NH. A case-control study of hidradenitis suppurativa in an STD population. Acta Derm Venereol. 1996;76(6):482–483. doi: 10.2340/0001555576482483. 8982418

[79] Zur Hausen H. Papillomaviruses in the causation of human cancers—a brief historical account. Virology. 2009;384(2):260–265. doi: 10.1016/j.virol.2008.11.046. 19135222

[80] Miller DL, Puricelli MD, Stack MS. Virology and molecular pathogenesis of HPV (human papillomavirus) associated oropharyngeal squamous cell carcinoma. Biochem J. 2012;443(2):339–353. doi: 10.1042/BJ20112017. 22452816 PMC3571652

[81] Scheinfeld N. A case of a patient with stage III familial hidradenitis suppurativa treated with 3 courses of infliximab and died of metastatic squamous cell carcinoma. Dermatol. Online J. 2014:20(3). 24656278

[82] Flores R, Lu B, Nielson C, et al. Correlates of human papillomavirus viral load with infection site in asymptomatic men. Cancer Epidemiol Biomarkers Prev. 2008;17(12):3573–3576. doi: 10.1158/1055-9965.EPI-08-0467. 19064573

[83] Li Z, Liu P, Wang Z, et al. Prevalence of human papillomavirus DNA and p16^INK4a^ positivity in vulvar cancer and vulvar intraepithelial neoplasia: a systematic review and meta-analysis. Lancet Oncol. 2023;24(4):403–414. doi: 10.1016/S1470-2045(23)00066-9. 36933562

[84] Krump NA, You J. Molecular mechanisms of viral oncogenesis in humans. Nat Rev Microbiol. 2018;16(11):684–698. doi: 10.1038/s41579-018-0064-6. 30143749 PMC6336458

[85] Mesri EA, Feitelson MA, Munger K. Human viral oncogenesis: a cancer hallmarks analysis. Cell Host Microbe. 2015;15(3):266–282. doi: 10.1016/j.chom.2014.02.011. PMC3992243 24629334

[86] Lawson JS, Glenn WK. Evidence for a causal role by human papillomaviruses in prostate cancer - a systematic review. Infect Agent Cancer. 2020;15:41. doi: 10.1186/s13027-020-00305-8. 32684946 PMC7359253

[87] Flood KS, Porter ML, Kimball AB. Biologic treatment for hidradenitis suppurativa. Am J Clin Dermatol. 2019;20(5):625–638. doi: 10.1007/s40257-019-00439-5. 31140067

[88] Giuffrida R, Cannavò SP, Coppola M, et al. Novel therapeutic approaches and targets for the treatment of hidradenitis suppurativa. Curr Pharm Biotechnol. 2021;22(1):59–72. doi: 10.2174/1389201021666200505100556. 32368973

[89] Mariette X, Matucci-Cerinic M, Pavelka K, et al. Malignancies associated with tumour necrosis factor inhibitors in registries and prospective observational studies: a systematic review and meta-analysis. Ann Rheum Dis. 2011;70(11):1895–1904. doi: 10.1136/ard.2010.149419. 21885875

[90] Tseng HW, Lu LY, Lam HC, et al. The influence of disease-modifying anti-rheumatic drugs and corticosteroids on the association between rheumatoid arthritis and skin cancer: a nationwide retrospective case-control study in Taiwan. Clin Exp Rheumatol. 2018;36(3):471–478. 29303707

[91] Lopez-Olivo MA, Tayar JH, Martinez-Lopez JA, et al. Risk of malignancies in patients with rheumatoid arthritis treated with biologic therapy: a meta-analysis. JAMA. 2012;308(9):898–908. Sep doi: 10.1001/2012.jama.10857. 22948700

[92] Thompson AE, Rieder SW, Pope JE. Tumor necrosis factor therapy and the risk of serious infection and malignancy in patients with early rheumatoid arthritis: a meta-analysis of randomized controlled trials. Arthritis Rheum. 2011;63(6):1479–1485. doi: 10.1002/art.30310. 21360522

[93] Balkwill F. TNF-a in promotion and progression of cancer. Cancer Metastasis Rev. 2006;25(3):409–416. doi: 10.1007/s10555-006-9005-3. 16951987

[94] Crisafulli S, Bertino L, Fontana A, et al. Incidence of skin cancer in patients with chronic inflammatory cutaneous diseases on targeted therapies: a systematic review and meta-analysis of observational studies. Front Oncol. 2021;11:687432. doi: 10.3389/fonc.2021.687432. 34150655 PMC8209509

[95] Kashii Y, Giorda R, Herberman RB, et al. Constitutive expression and role of the TNF family ligands in apoptotic killing of tumor cells by human NK cells. J Immunol. 1999;163(10):5358–5366. doi: 10.4049/­jimmunol.163.10.5358. 10553060

[96] Prévost-Blondel A, Roth E, Rosenthal FM, et al. Crucial role of TNF-alpha in CD8 T cell-mediated elimination of 3LL-A9 Lewis lung carcinoma cells in vivo. J Immunol. 2000;164(7):3645–3651. doi: 10.4049/jimmunol.164.7.3645. 10725721

[97] Calzascia T, Pellegrini M, Hall H, et al. TNF-alpha is critical for antitumor but not antiviral T cell immunity in mice. J Clin Invest. 2007;117(12):3833–3845. doi: 10.1172/JCI32567. 17992258 PMC2066188

[98] Askling J, Fahrbach K, Nordstrom B, et al. Cancer risk with tumor necrosis factor alpha (TNF) inhibitors: meta-analysis of randomized controlled trials of adalimumab, etanercept, and infliximab using patient level data. Pharmacoepidemiol Drug Saf. 2011;20(2):119–130. doi: 10.1002/pds.2046. 21254282

[99] Wang JL, Yin WJ, Zhou LY, et al. Risk of non-melanoma skin cancer for rheumatoid arthritis patients receiving TNF antagonist: a systematic review and meta-analysis. Clin Rheumatol. 2020;39(3):769–778. doi: 10.1007/s10067-019-04865-y. 31823140

[100] Wetzman A, Lukas C, Gaujoux-Viala C, et al. Risk of cancer after initiation of targeted therapies in patients with rheumatoid arthritis and a prior cancer: systematic review with meta-analysis. Arthritis Care Res (Hoboken). 2023;75(2):260–271. doi: 10.1002/acr.24784. 34549898

[101] Geller S, Xu H, Lebwohl M, et al. Malignancy risk and recurrence with psoriasis and its treatments: a concise update. Am J Clin Dermatol. 2018;19(3):363–375. doi: 10.1007/s40257-017-0337-2. 29260411 PMC5948118

[102] Geborek P, Bladström A, Turesson C, et al. Tumour necrosis factor blockers do not increase overall tumour risk in patients with rheumatoid arthritis, but may be associated with an increased risk of lymphomas. Ann Rheum Dis. 2005;64(5):699–703. doi: 10.1136/ard.2004.030528. 15695534 PMC1755491

[103] Wolfe F, Michaud K. Lymphoma in rheumatoid arthritis: the effect of methotrexate and anti-tumor necrosis factor therapy in 18,572 patients. Arthritis Rheum. 2004;50(6):1740–1751. doi: 10.1002/art.20311. 15188349

[104] Haynes K, Beukelman T, Curtis JR, SABER Collaboration, et al. Tumor necrosis factor α inhibitor therapy and cancer risk in chronic immune-mediated diseases. Arthritis Rheum. 2013;65(1):48–58. doi: 10.1002/art.37740. 23055441 PMC3778442

[105] Setoguchi S, Solomon DH, Weinblatt ME, et al. Tumor necrosis factor alpha antagonist use and cancer in patients with rheumatoid arthritis. Arthritis Rheum. 2006;54(9):2757–2764. doi: 10.1002/art.22056. 16947774

[106] Jung JM, Kim YJ, Chang SE, et al. Cancer risks in patients with psoriasis administered biologics therapy: a nationwide population-based study. J Cancer Res Clin Oncol. 2023;149(19):17093–17102. doi: 10.1007/s00432-023-05387-6. 37755577 PMC11796492

[107] Arcuri D, Kaouache M, Lagacé F, et al. A case-control pharmacovigilance study of TNF-alpha inhibitors and interleukin inhibitors on tuberculosis, Candida, lymphoma and suicidality using the FAERS database (2014-2020). J Am Acad Dermatol. 2023;89(3):619–621. doi: 10.1016/j.jaad.2023.05.041. 37245836

[108] Kimball AB, Jemec GBE, Alavi A, et al. Secukinumab in moderate-to-severe hidradenitis suppurativa (SUNSHINE and SUNRISE): week 16 and week 52 results of two identical, multicentre, randomised, placebo-controlled, double-blind phase 3 trials. Lancet. 2023;401(10378):747–761. doi: 10.1016/S0140-6736(23)00022-3. 36746171

[109] Snyder CL, Gibson RS, Porter ML, et al. Secukinumab in the treatment of hidradenitis suppurativa. Immunotherapy. 2023;15(17):1449–1457. doi: 10.2217/imt-2023-0103. 37840286

[110] Bilal J, Berlinberg A, Riaz IB, et al. Risk of infections and cancer in patients with rheumatologic diseases receiving interleukin inhibitors: a systematic review and meta-analysis. JAMA Netw Open. 2019;2(10):e1913102. doi: 10.1001/jamanetworkopen.2019.13102. 31626313 PMC6813598

[111] Yon JR, Son JD, Fredericks C, et al. Marjolin’s ulcer in chronic hidradenitis suppurativa: a rare complication of an often neglected disease. J Burn Care Res. 2017;38(2):121–124. doi: 10.1097/BCR.0000000000000399. 27380120

[112] Huang C, Lai Z, He M, et al. Successful surgical treatment for squamous cell carcinoma arising from hidradenitis suppurativa: A case report and literature review. Medicine (Baltimore). 2017;96(3):e5857. doi: 10.1097/MD.0000000000005857. 28099342 PMC5279087

[113] Chang JB, Kung TA, Cederna PS. Acute Marjolin’s ulcers: A nebulous diagnosis. Ann Plast Surg. 2014;72(5):515–520. doi: 10.1097/SAP.0000000000000134. 24691319

[114] Beard CJ, Gathings RM, Bandino JP. Exophytic mass arising within hidradenitis suppurativa: answer. Am J Dermatopathol. 2019;41(3):235–236. doi: 10.1097/DAD.0000000000001064. 30801344

[115] Foster DS, Jones RE, Ransom RC, et al. The evolving relationship of wound healing and tumor stroma. JCI Insight. 2018;3(18):e99911. doi: 10.1172/jci.insight.99911. 30232274 PMC6237224

[116] Öhlund D, Elyada E, Tuveson D. Fibroblast heterogeneity in the cancer wound. J Exp Med. 2014;211(8):1503–1523. doi: 10.1084/jem.20140692. 25071162 PMC4113948

[117] Darby I, Skalli O, Gabbiani G. Alpha-smooth muscle actin is transiently expressed by myofibroblasts during experimental wound healing. Lab Invest. 1990;63(1):21–29. 2197503

[118] Desmoulière A, Guyot C, Gabbiani G. The stroma reaction myofibroblast: a key player in the control of tumor cell behavior. Int J Dev Biol. 2004;48(5–6):509–517. doi: 10.1387/ijdb.041802ad. 15349825

[119] Rønnov-Jessen L, Petersen OW. Induction of alpha-smooth muscle actin by transforming growth factor-beta 1 in quiescent human breast gland fibroblasts. Implications for myofibroblast generation in breast neoplasia. Lab Invest. 1993;68(6):696–707. 8515656

[120] Bechara FG, Sand M, Skrygan M, et al. Acne inversa: evaluating antimicrobial peptides and proteins. Ann Dermatol. 2012;24(4):393–397. doi: 10.5021/ad.2012.24.4.393. 23197903 PMC3505768

[121] Kim J, Lee J, Li X, et al. Single-cell transcriptomics suggest distinct upstream drivers of IL-17A/F in hidradenitis versus psoriasis. J Allergy Clin Immunol. 2023;152(3):656–666. doi: 10.1016/j.jaci.2023.05.012. 37271319 PMC11057969

[122] Jiménez-Gallo D, de la Varga-Martínez R, Ossorio-García L, et al. The clinical significance of increased serum proinflammatory cytokines, C-reactive protein, and erythrocyte sedimentation rate in patients with hidradenitis suppurativa. Mediators Inflamm. 2017;2017:2450401–2450408. doi: 10.1155/2017/2450401. 28769536 PMC5523401

[123] Hessam S, Gambichler T, Höxtermann S, et al. Frequency of circulating subpopulations of T-regulatory cells in patients with hidradenitis suppurativa. J Eur Acad Dermatol Venereol. 2020;34(4):834–838. doi: 10.1111/jdv.16071. 31721309

[124] Lee HJ, Hong YJ, Kim M. Angiogenesis in chronic inflammatory skin disorders. Int J Mol Sci. 2021;22(21):12035. doi: 10.3390/ijms222112035. 34769465 PMC8584589

[125] Jones D, Banerjee A, Berger PZ, et al. Inherent differences in keratinocyte function in hidradenitis suppurativa: evidence for the role of IL-22 in disease pathogenesis. Immunol Invest. 2018;47(1):57–70. doi: 10.1080/08820139.2017.1377227. 28972431 PMC6207448

[126] Sinha S, Su S, Workentine M, et al. Transcriptional analysis reveals evidence of chronically impeded ECM turnover and epithelium-to-mesenchyme transition in scar tissue giving rise to Marjolin’s ulcer. J Burn Care Res. 2017;38(1):e14–e22. doi: 10.1097/BCR.0000000000000432. 27679957

[127] Schlapbach C, Hänni T, Yawalkar N, et al. Expression of the IL-23/Th17 pathway in lesions of hidradenitis suppurativa. J Am Acad Dermatol. 2011;65(4):790–798. doi: 10.1016/j.jaad.2010.07.010. 21641076

[128] Hessam S, Sand M, Gambichler T, et al. Interleukin-36 in hidradenitis suppurativa: evidence for a distinctive proinflammatory role and a key factor in the development of an inflammatory loop. Br J Dermatol. 2018;178(3):761–767. doi: 10.1111/bjd.16019. 28975626

[129] Del Duca E, Morelli P, Bennardo L, et al. Cytokine pathways and investigational target therapies in hidradenitis suppurativa. Int J Mol Sci. 2020;21(22):8436. doi: 10.3390/ijms21228436. 33182701 PMC7696820

[130] Qi Y, Liao B, Chen J, et al. Diagnostic value of IL-22, IL-23, and IL-17 for NK/T cell lymphoma. Cell Mol Biol (Noisy-Le-Grand). 2023;69(3):150–155. doi: 10.14715/cmb/2023.69.3.22. 37300674

[131] Rothman N, Skibola CF, Wang SS, et al. Genetic variation in TNF and IL10 and risk of non-Hodgkin lymphoma: a report from the InterLymph Consortium. Lancet Oncol. 2006;7(1):27–38. doi: 10.1016/S1470-2045(05)70434-4. 16389181

[132] Hu Y, Xu D, Xia H, et al. Associations of IL-17A -197G/A and IL-17F 7488T/C polymorphisms with cancer risk in asians: An updated meta-analysis from 43 studies. Gene. 2021;804:145901. doi: 10.1016/j.gene.2021.145901. 34403774

[133] Liu XH, Dai ZM, Kang HF, et al. Association of IL-23R polymorphisms (rs6682925, rs10889677, rs1884444) with cancer risk: A PRISMA-compliant meta-analysis. Medicine (Baltimore). 2015;94(52):e2361. doi: 10.1097/MD.0000000000002361. 26717375 PMC5291616

[134] Queen D, Ediriweera C, Liu L. Function and regulation of IL-36 signaling in inflammatory diseases and cancer development. Front Cell Dev Biol. 2019;7:317. doi: 10.3389/fcell.2019.00317. 31867327 PMC6904269

[135] Li C, Xu MM, Wang K, et al. Macrophage polarization and meta-inflammation. Transl Res. 2018;191:29–44. doi: 10.1016/j.trsl.2017.10.004. 29154757 PMC5776711

[136] Mintoff D, Benhadou F, Pace NP, et al. Metabolic syndrome and hidradenitis suppurativa: epidemiological, molecular, and therapeutic aspects. Int J Dermatol. 2022;61(10):1175–1186. doi: 10.1111/ijd.15910. 34530487

[137] Mintoff D, Agius R, Benhadou F, et al. Obesity and hidradenitis suppurativa: targeting meta-inflammation for therapeutic gain. Clin Exp Dermatol. 2023;48(9):984–990. doi: 10.1093/ced/llad182. 37171791

[138] Gambichler T, Hessam S, Cramer P, et al. Complete blood collection-based systemic inflammation biomarkers for patients with hidradenitis suppurativa. J Eur Acad Dermatol Venereol. 2022;36(9):1593–1596. doi: 10.1111/jdv.18175. 35462426

[139] Hai-Jing Y, Shan R, Jie-Qiong X. Prognostic significance of the pretreatment pan-immune-inflammation value in cancer patients: an updated meta-analysis of 30 studies. Front Nutr. 2023;10:1259929. doi: 10.3389/fnut.2023.1259929. 37850085 PMC10577316

